# Simulative Minimization of Mass Transfer Limitations Within Hydrogel-Based 3D-Printed Enzyme Carriers

**DOI:** 10.3389/fbioe.2020.00365

**Published:** 2020-04-28

**Authors:** Barbara Schmieg, Mai Nguyen, Matthias Franzreb

**Affiliations:** Karlsruhe Institute of Technology, Institute of Functional Interfaces, Karlsruhe, Germany

**Keywords:** enzyme immobilization, hydrogel, mass transfer limitation, effectiveness factor, Thiele modulus, 3D-printing

## Abstract

In biotechnology, immobilization of functional reactants is often done as a surface immobilization on small particles. Examples are chromatography columns and fixed-bed reactors. However, the available surface for immobilization is directly linked to particle diameter and bed porosity for these systems, leading to high backpressure for small particle sizes. When larger molecules, such as enzymes are immobilized, physical entrapment within porous materials like hydrogels is an alternative. An emerging technique for the production of geometrically structured, three-dimensional and scalable hollow bodies is 3D-printing. Different bioprinting methods are available to produce structures of the desired size, resolution and solids content. However, in case of entrapped enzymes mass transfer limitations often determine the achievable reactivities. With increasing complexity of the system, for example a fixed-bed reactor, 3D-simulation is indispensable to understand the local reaction conditions to be able to highlight the optimization potential. Based on experimental data, this manuscript shows the application of the dimensionless numbers effectiveness factor and Thiele modulus for the design of a 3D-printed flow-through reactor. Within the reactor, enzymes are physically entrapped in 3D-printed hydrogel lattices. The local reaction rate of the enzymes is directly dependent on the provided substrate amount at the site of reaction which is limited by the diffusion properties of the hydrogel matrix and the diffusion distance. All three parameters can be summed up by one key figure, the Thiele modulus, which, in short, quantifies mass transfer limitations of a catalytic system. Depending on the rate of the enzymatic reaction in correlation to the diffusional transport, mass transfer limitations will shift the optimum of the system, favoring slow enzyme kinetics and small diffusion distances. Comparison with the enzymatic reaction rate in solution yields the effectiveness factor of the system. As a result, the optimization potential of varying the 3D-printed geometries or the reaction rate within the experimentally available design space can be estimated.

## Introduction

Continuous reactors for heterogeneous catalysis prevalently consist of tube systems filled with porous particles (Manjón et al., 1987; Khalilpour and Roostaazad, 2008). The enzymes are immobilized onto the particle surfaces or within the pores, which makes them accessible by the fluid phase containing the substrate(s). The specific surface available for the enzyme fixation is mainly determined by the size of the particles itself and their material-dependent inherent porosity (Munirathinam et al., 2015). Concerning the packing density, the maximum porosity is about 0.45 for the fluidic proportion of the reactor in case of perfectly spherical particles (Jungbauer and Hahn, 2008). Irregular particles will decrease the interstitial fluid volume and lead to an increasing backpressure. As an alternative for higher flowrates, monoliths have been widely investigated (Pfaunmiller et al., 2013; Sandig and Buchmeiser, 2016; Masini and Svec, 2017; Onbas and Yesil-Celiktas, 2019). However, they are mainly applied in small analytic systems, as limitations arise in scale-up (Jungbauer and Hahn, 2008; Tourlomousis and Chang, 2016).

As a facile method for the fabrication of highly porous, intricate geometries, 3D-printing has been brought up. Fee et al. proposed the idea to broaden the scope for chromatographic resin from randomly shaped particles and spheres to regular geometric patterns, which can be 3D-printed (Fee et al., 2014). The same group has shown the potential of the concept by simulation of the dispersive behavior which exceeds randomly packed columns (Nawada et al., 2017) and the proof of concept by printing acrylonitrile-butadiene-styrene (Fee et al., 2014) and a polyethylene glycol diacrylate-based anion exchange resin via digital light processing (Simon and Dimartino, 2018).

3D-printing enables the decoupling of the parameters bed porosity and material properties. The interstitial volume of the printed structures can be chosen freely, as one material can be printed into (almost) any desired geometry. If the mechanical stability is weak, permanent support structures can be added, leaving the resolution of the printer as the only process limiting parameter. The inherent porosity of the material itself is governed its composition, which has to be suitable for 3D-printing. To broaden the scope of applications functionalization of the 3D-printed geometry can be done as a post-processing step (Kazenwadel et al., 2016).

In our previous publication (Schmieg et al., 2019), we presented a 3D-printed fixed-bed reactor with 3D-printed hydrogel lattices for the physical entrapment of enzymes. Feasibility of the immobilization was shown for three enzymes, two of them were unpurified crude cell extracts. Due to the modular concept, scale-up can be done via elongation of the 3D-printed reactor chamber and the number of hydrogel lattices inserted.

For the optimization of processes conducted in such a 3D-printed fixed-bed reactor, this manuscript shows a simulative approach to find optimal geometric parameters for the given enzymatic kinetics of β-Galactosidase and the diffusional behavior of the used polyethylene glycol diacrylate-based hydrogel (Schmieg et al., 2018). Boundary conditions for experimental optimization are the limitations of the extrusion-based 3D-printer, for example the applicability of the printed material for extrusion. Furthermore, long, continuous lines are preferred over dots for fast and exact printing, so lattice structures are easiest to produce. Strand thickness for stable 3D-printed lattices is limited to dimensions of more than 400–500 μm for extrusion-based systems.

The underlying mathematical equations of the process are diffusional mass transfer limitations (Equation 1; Sharma, 2010) and enzymatic reaction kinetics. These are linked by the dimensionless numbers Thiele modulus and effectiveness factor, which will be presented in the following section.

(1) $$\frac{\partial c}{\partial t}=D\,\frac{\partial^{2}c}{\partial x^{2}}$$

The Thiele modulus ϕ (Equation 2) is a dimensionless number that relates the geometric distance of diffusive transport *L*, the reaction rate *k* of the catalyst with a reaction order of *n* and the effective diffusion coefficient *D*_*eff*_ of the matrix material where the diffusive transport takes place (Petersen, 1965).

(2) $$\phi =L\cdot \sqrt{\frac{k\cdot c_{\mathrm{substrate}}^{n-1}}{D_{\mathrm{eff}}}}$$

The effectiveness factor is the ratio between enzymatic activity monitored in immobilized state compared to free solution (Equation 3). It is a key figure that can assume values between 0 and 1 that define the efficiency of the technically more complex setup. The factor gains importance when the economic efficiency of a plant or process is calculated.

The effectiveness factor of immobilized enzymes can be characterized via the Thiele modulus. For the simple geometries sphere, cylinder and infinite slab, analytical solutions are available, as shown in Equations (4)–(6) (Petersen, 1965). Hereby, Bessel functions of the first kind *J*_0_(*x*) and *J*_0_(*x*) are used in the equation for the cylinder.

$$\eta_{\mathrm{catalytic}}=\frac{\mathrm{reaction}\,\mathrm{rate}\,\left(\mathrm{Enzyme}\,\mathrm{in}\,\mathrm{Hydrogel}\right)}{\mathrm{reaction}\,\mathrm{rate}\,\left(\mathrm{Enzyme}\,\mathrm{in}\,\mathrm{Solution}\right)}$$

(3) $$=\frac{\frac{m_{\mathrm{product},\mathrm{Hydrogel}}}{m_{\mathrm{Enz},\mathrm{Hydrogel}}}}{\frac{m_{\mathrm{product},\mathrm{Solution}}}{m_{\mathrm{Enz},\mathrm{Solution}}}}$$

(4) $$\eta_{\mathrm{sphere},1\,\mathrm{st}\,\mathrm{order}}=\frac{3}{\phi }\cdot \left(\frac{1}{\tanh\,\left(\phi \right)}-\frac{1}{\phi }\right)$$

(5) $$\eta_{\mathrm{cylinder},1\,\mathrm{st}\,\mathrm{order}}=\frac{2}{\phi }\cdot \left(\frac{{\mathrm{iJ}}_{1}\,\left(i\,\phi \right)}{J_{0}\,\left(i\,\phi \right)}\right)$$

(6) $$\eta_{\mathrm{infinite}\,\mathrm{slab},1\,\mathrm{st}\,\mathrm{order}}=\frac{\tanh\,\left(\phi \right)}{\phi }$$

Diffusional limitations for catalytic systems were already described by a number of authors for microfluidic systems or for theoretical descriptions of scale-up strategies (AbuReesh, 1997; Jodlowski et al., 2017; Baronas et al., 2018).

For example, Zohdi-Fasaei et al. (2016) discuss the optimal size of silica-supported metal catalyst pellets based on the Thiele modulus, stating that diffusion limits the kinetics at pellet sizes larger than 0.5 mm. Manjón et al. (1987) analyzed the effectiveness factor of a particle-filled packed bed reactor in dependence of substrate and product concentration for an enzymatic reaction. This was deepened by Baronas et al. (2018) for an assumed dimensionless reactor filled with enzymes immobilized in porous particles.

All studies show that entrapment favors slow reaction kinetics, as higher reaction rates will cause the consumption of the substrate at the outer boundaries of the catalytically active geometries, as (Garcia-Galan et al., 2011) sums up. 3D-printing offers the flexibility in geometry to adapt the process setup to intricate geometries for an improved utilization of the catalytic reaction potential. With a basic understanding of how the reaction rate governs the process performance, transfer to various enzymatic systems in facilitated.

## Materials

### Experimental Data

**FIGURE 1:**
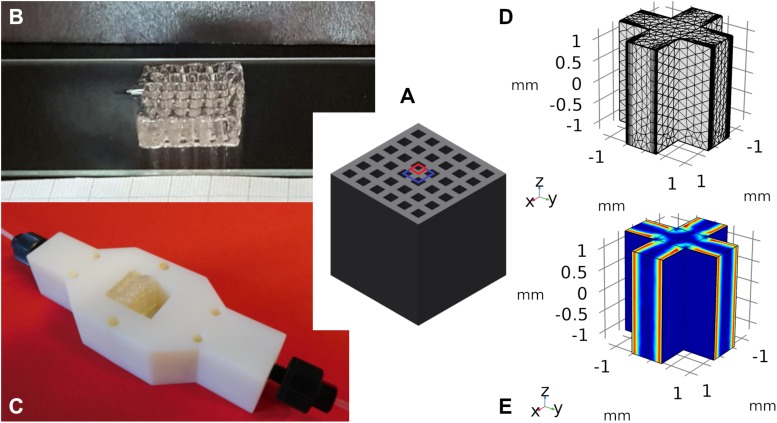
3D-printed lattices for the physical entrapment of enzymes. **(A)** Schematic view of the printed lattice geometry, with dimensions of 13 × 13 × 13 mm. The red quadrilateral shows the 2D unit cell used in the modeling in section unit cell porosity optimization, the blue quadrilateral shows the cross-section of the 3D unit cell used in the modeling of section conversion in the 3D-system. **(B)** Hydrogel lattice structure produced by extrusion-based 3D-printing. **(C)** Lattices are inserted into a 3D-printed reactor geometry with pore axis parallel to the flow direction. **(D)** The unit cell of the lattice, here called “cross” and its mesh for COMSOL simulation. **(E)** Concentration profile within an exemplary cross structure.

The simulation is based on experimental data for the immobilization of β-Galactosidase from *Aspergillus oryzae* in 3D-printed hydrogel lattices, which were already published by our group (Schmieg et al., 2019). In short, the hydrogel consists of a heterogeneous mixture of polyethylene-glycol diacrylate polymer chains as well as colloidal silicate particles and enzymes (Schmieg et al., 2018). It was 3D-printed with an extrusion printing system into rectangular hydrogel lattices (Figures 1A,B) with outer dimensions of 13 × 13 × 3 mm. These were inserted in a tailor-made, 3D-printed reactor module with a 3 ml chamber volume and a square cross-section (Figure 1C). The fixed-bed reactors showed stable operation over at least three days. On top of that, batch reaction kinetics of suspended hydrogel lattices were done to estimate the enzymatic conversion by entrapped enzymes in comparison to that of freely dissolved enzyme.

From the experiments, the kinetic parameters for the enzymatic reaction were gained. For first-order Michaelis-Menten kinetics, a value of 0.13 mmol l^–1^ min^–1^ was calculated for v_*max*_, which defines the maximum reaction rate of the enzyme in case of substrate excess. K_*m*_, the substrate concentration which is applied to reach 0.5⋅*v*_*max*_, is 1.4 mmol l^–1^ (Schmieg et al., 2019). The hydrogel is approximated as a homogenous material with an effective diffusion coefficient of 3E–12 m^2^ s^–1^ for the β-Galactosidase substrate o-nitrophenyl-β-D-galactopyranoside (Schmieg et al., 2018). No additional models for the internal hydrogel structure, for example parameters for polymer tortuosity and mesh size were used.

### Process Simulation

Process simulation was done with the COMSOL Multiphysics software (V 5.4) (COMSOL AB, Stockholm, Sweden). The software was running on a Desktop computer (CPU: i7-7700@3.6 GHz, quad core, 8 threads; GPU: Nvidia sQuadro P600; RAM: 32 Gb DDR3; System: windows 10 64 bit, version 1803). Exported data were analyzed with Excel 2013 (Microsoft Corporation, Redmond, Washington, United States). The enzymatically catalyzed reaction within the hydrogel geometry was simulated with the physics module “Transport of Diluted Species in Diluted Media” of COMSOL. Equations for enzymatic reaction, diffusive mass transfer within the hydrogel matrix and laminar flow and convection within the fluid phase were used. To simplify the process, it was assumed that no adsorption of substrate or product molecules takes place within the hydrogel. The used global parameters are allocated in Table 1 (for details see Supporting Information files). Their calculation will be explained in the following sections.

**TABLE 1:** Global parameters and variables for the COMSOL simulation.

Parameter	Value	Unit	Annotations
Temperature	293.15	K	Global parameter for all experiments
D_*eff,hydrogel*_	3E–12	m^2^s^–1^	Effective diffusion coefficient in the hydrogel based on experimental data
D_*solution*_	1E–9	m^2^s^–1^	Diffusion coefficient within the liquid phase
c_*i,0,hydrogel*_	0	mol m^–3^	Initial conditions for reactants *i* in the hydrogel
c_*i,solution*_	Variable	mol m^–3^	Concentration of species *i* in the solution around the hydrogel
c_*A*_	Variable	mol m^–3^	Local concentration of the substrate.
c_*B*_	Variable	mol m^–3^	Local concentration of the product.
v_*max*_	Variable	mol s^–1^ m^–3^	Maximum reaction rate according to Michaelis-Menten kinetics
K_*m*_	1.4	mol m^–3^	Michaelis-Menten parameter based on experimental data
k	Variable	s^–1^	Reaction rate constant for first order kinetics
r_*A*_	$\frac{-v_{m\,a\,x}\cdot c_{A}}{K_{m}+c_{A}}$	mol s^–1^ m^–3^	Reaction rate for the consumption of substrate A according to Michaelis-Menten kinetics.
r_*B*_	$\frac{v_{m\,a\,x}\cdot c_{B}}{K_{m}+c_{B}}$	mol s^–1^ m^–3^	Reaction rate for the formation of product B according to Michaelis-Menten kinetics
k_*eff*_	$\frac{v_{m\,a\,x}}{K_{m}+c_{A,s\,o\,l\,u\,t\,i\,o\,n}}$	s^–1^	Apparent reaction constant for the expression of the initial Michaelis-Menten reaction rate as a 1st order kinetics Similar to the definition of the Weisz modulus Neira and Herr, 2017
a_*unit cell*_	Variable	m	Edge length of the unit cell

*Detailed settings of COMSOL are available in the Supporting Information.*

#### Model Structures

To approximate the measured batch data, a simple unit cell to simulate the process was desired. In experiments, hydrogel lattices with a strand diameter of ∼0.8 mm were 3D-printed (Figures 1A,B), as 3D-printed structures exhibit the highest printing fidelity when repetitive, simple geometries are used. Furthermore, extrusion-based printers can produce continuous strands of material best. Aligning and stacking horizontal and vertical hydrogel strands will result in very stable hydrogel geometries with regular flow patterns. For a better understanding of the effect of the used shape, basic geometries like sphere, and cylinder as well as the simplest representation of the 3D-printed hydrogel structure, a cross (Figures 1D,E), were chosen to validate the initial model of the enzymatic reaction within the hydrogel strand. To shorten the computing time, the unit cells were split along the symmetry axes of the geometry and some simulations were done with a quarter of the unit cell. This can be done, because the cross-shaped hydrogel rods forming the repetitive unit cells of the 3D-printed structure show two middle planes of symmetry with the planes being parallel to the flow direction. Along these symmetry planes no concentration gradients and therefore no molecule fluxes exist.

#### Physical Parameters and Assumptions

Within the “Transport of Diluted Species in Diluted Media” physics module, isotropic diffusion is set as the only driving force for mass transfer in the hydrogel. Transport via convection was activated in the liquid phase in addition to the transport via diffusion applying a diffusion coefficient of D_*solution*_ = 1E–9 m^2^/s. The initial concentrations within the hydrogel are all set to zero. For the enzymatic reaction, two kinetic models were used. First order kinetics is abundantly used in simulation of catalytic processes. Moreover, in this case analytical solutions for the Thiele modulus are available, which can be used to validate the simulation model (see section model validation). As the cleavage of o−nitrophenyl−β−D−galactopyranoside by β-Galactosidase obeys first order Michaelis-Menten kinetics (Schmieg et al., 2019), the Thiele modulus was also calculated for this model. In contrast to first order kinetics, here the reaction order is depending on the substrate concentration. In case of a limited substrate amount, reaction order obeys a pseudo first-order reaction and approaches a zero order reaction in case of substrate excess. Enzymes entrapped in the hydrogel were assumed to have the same intrinsic activity as in solution. As a result, mass transfer limitation is the only physical process responsible for reducing the effectiveness factor of the immobilized enzymes.

### Model Validation

The validation of the multiphysics model was executed for the geometries of a sphere and a cylinder in combination with first order kinetics. With the resulting reaction rate constant k in the unit s^–1^, the Thiele modulus ϕ is independent of the substrate concentration as shown in Equation (7). For Michaelis-Menten kinetics, simplification to insert the first order reaction rate constant into the equation of the Thiele modulus was done as stated in Equation (8), similar to a calculation that was shown in Neira and Herr (2017).

(7) $$\phi =L\cdot \sqrt{\frac{k}{D}}$$

(8) $$\phi =L\cdot \sqrt{\frac{k_{\mathrm{eff}}}{D}}=L\cdot \sqrt{\frac{\frac{v_{\max}}{K_{m}+c_{A,\mathrm{solution}}}}{D}}$$

Analytical solutions of the effectiveness factor as a function of the Thiele modulus [see Equations (4)–(6)] are available for the simple geometries sphere, (infinte) cylinder and infinite slab, so the numeric model should generate identical solutions. A distance of 400 μm was used for the radius of the sphere and the cylinder. Accordingly, the hydrogel strand thickness of the simulated unit cell structure was 800 μm. By using symmetry conditions at the apparent cut faces of the cylinder and cross geometries infinite structures could be approximated. A constant substrate concentration at the outer edges of the hydrogel structures (c_*A*,__*solution*_) was used to model substrate excess. The mesh was built based on the “normal size” setting of COMSOL with the addition of eight boundary layers (Boundary layer stretching factor 1.2, thickness of the first layer of 0.01 mm). A stationary study was conducted. For a variation of the Thiele modulus via the reaction rate, a range of k ∈ [1E–6 s^–1^, 0.1 s^–1^] and v_*max*_ ∈ [0.01 mol m^–3^ s^–1^, 10 mol m^–3^ s^–1^] were chosen for first order kinetics and Michaelis-Menten kinetics respectively with logarithmic interpolation between the values. Tested substrate concentrations c_*A,bulk*_ were 0.5, 2, and 20 mol m^–3^. With a parameter sweep, the effectiveness factor for the parameter combinations was calculated.

### Unit Cell Porosity Optimization

With the aim of optimizing a 3D-printed lattice structure in terms of productivity, a parameter that can be varied relatively easy in 3D-printing experiments is the hydrogel strand thickness. As a first approximation, a 2D unit cell was designed. The 2D unit cell represents a quarter of the cross-section through a hydrogel cross (Figure 1A, red quadrilateral). For a given unit cell edge length of 1.5 mm, the hydrogel strand thickness and the substrate concentration were varied in the range between 0.04 and 1.46 mm respectively 2 and 42 mM. As the unit cell is symmetrical, only a quarter of it was used for the simulation in COMSOL to save computing time.

A stationary study was conducted that included the diffusive transport of the substrate from the liquid phase into the hydrogel strand, the enzymatic reaction according to Michaelis-Menten kinetics and diffusion of product within the unit cell. The elements of the mesh had a size between 2.4E–5 m and 4E–2 m. On the interphase from liquid phase to hydrogel, eight boundary layers (Boundary layer stretching factor 1.4, thickness of the first layer of 2 μm) were introduced. The evaluation of the resulting concentration profiles within the hydrogel regarding their potential to generate the desired product was conducted by three approaches. First, the mean value of the substrate concentration c_*A*_ over the hydrogel area of the unit cell was calculated and plotted against the hydrogel strand thickness within the given unit cell. Furthermore, a better approximation of the formation of product B within the unit cell was calculated by using the constant pseudo-first order reaction rate *k*_*eff*_ and calculating the local reaction rate by first order kinetics in each point of the hydrogel. The average first order reaction rate within the unit cell is then derived by the surface integral of the local reaction rate divided by the unit cell area (Equation 9). Finally, the true enzymatic reaction rate within the unit cell was calculated applying Michaelis-Menten kinetics (Equation 10) for the calculation of the local reaction rate, followed by the same surface integral and normalization. With these calculations, differences resulting from the assumptions regarding enzyme kinetics and the influence of the substrate concentration will be highlighted.

(9) $$r_{B\,\mathrm{first}\,\mathrm{order}}=\frac{1}{A_{\mathrm{unit}\,\mathrm{cell}}}\,c_{A}\,k_{e\,f\,f}\,d\,A_{h\,y\,d\,r\,o\,g\,e\,l}$$

(10) $$r_{B\,\mathrm{Michaelis}\,\mathrm{Menten}}=\frac{1}{A_{\mathrm{unit}\,\mathrm{cell}}}\,\frac{v_{m\,a\,x}\cdot c_{A}}{K_{m}+c_{A}}\,d\,A_{h\,y\,d\,r\,o\,g\,e\,l}$$

### Assessment of the Three-Dimensional Reactor

Within the enzymatic fixed bed reactor, the available substrate concentration will decrease as the substrate solution traverses the reactor. As a result, the potentially produced amount of product per cross section that was calculated in the 2D model will differ across the reactor length and necessitates calculation within a 3D-unit cell. On top of that, time-dependent behavior of substrate conversion is more pronounced in the 3D-simulation than in the 2D case, and the start-up phase of the reactor experiment can be described.

For the three-dimensional case, a length *L*_*reactor*_ of 15 mm for the reactor chamber was used, which corresponds the experimental geometry used in Schmieg et al. (2019). The edge length of the unit cell was chosen accordingly, it amounts to 1.5 mm. The volumetric flow rate $\dot{V}$ = 3 mL passes through 64 unit cells within the 3D-printed reactor chamber, so the linear flow rate *u*_*water*_ equals 8.3E–10 ms^–1^. At the reactor entry, the substrate concentration *c*_*A,solution*_ was chosen in the range of 2–42 mM and the respective product formation was estimated according to Equation (11) for hydrogel strand thicknesses between 0.05 and 1.45 m.

(11) $$r_{B\,\mathrm{Michaelis}\,\mathrm{Menten}}=\frac{1}{V_{\mathrm{unit}\,\mathrm{cell}}}\,∭\frac{v_{\max}\cdot c_{A}}{K_{m}+c_{A}}\,{\mathrm{dV}}_{\mathrm{hydrogel}}$$

Additionally, the time-dependent substrate and product concentrations in the fluid phase flowing out of the reactor were plotted for three representative hydrogel thicknesses of 100, 250, and 700 μm and a substrate concentration of 2.2 mM. To visualize the temporal and spatial changes of the product concentration, color maps are shown for the same hydrogel strand thicknesses and exemplary times of 1, 3 h and the stationary state after 48 h duration. Thereby, the unit cell sectional view was generated through the center of the hydrogel strand over the length of the reactor, showing the local deviations within the hydrogel. Concentration profiles in the stationary phase after 48 h show the cross-section at the exit of the reactor, where local concentrations in the hydrogel and the fluid channel can be distinguished.

For the stationary and the time-dependent study of the process, the COMSOL physics module “Transport of Diluted Species in Diluted Media” and a mesh with eight boundary layers were generated analogous to section unit cell porosity optimization.

## Results

### Model Validation

The Thiele modulus is a key factor for describing the limitations brought upon a catalytic pellet by the diffusion coefficient. Evaluation of the effectiveness factor against the Thiele modulus is a common technique in process engineering when systems are scaled up or down according to dimensionless numbers (Petersen, 1965; Weisz, 1973). In the enzymatic reactor system discussed here, enzyme variants or hydrogel compositions can be screened and optimized experimentally. The variation of single parameters in a simulation gives insights into the extent of optimization potential of the system.

**FIGURE 2:**
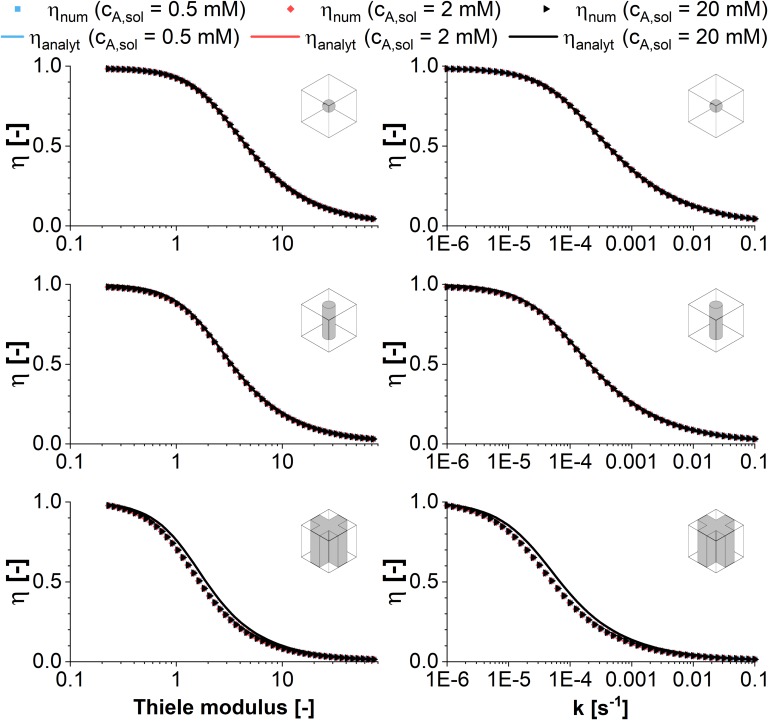
Comparison of analytical (symbols) and numeric (lines) solutions of the effectiveness factor plotted vs. the Thiele modulus and the reaction rate constant according to first order kinetics. The effectiveness factor is calculated for the model structures sphere, infinite cylinder, and cross-shaped infinite rod for three exemplary substrate concentrations of 0.5, 2, and 20 mM. Taking into account the experimental diffusion coefficient of 3E–12 m^2^s^–1^, the resulting Thiele moduli vary between 0.23 and 73.

First order kinetics were used to validate the COMSOL model for the geometries sphere, cylinder and cross. According to Equation (2), the Thiele modulus is independent of the used substrate concentration in the fluid phase in that case. Consequently, the effectiveness factor shows a sigmoid decrease for increasing Thiele moduli, which is displayed in Figure 2. The exact slope is depending on the model geometry and thus, on the exact surface to volume ratio. As the Thiele modulus was varied by a parameter sweep of k, the reaction rate constant of the first order kinetics, it can be stated that the higher the reaction velocities the lower the effectiveness factor and the productivity of immobilized catalysts. Within the hydrogel, the reaction of substrate takes place much faster than its diffusional propagation. Part of the enzymes at the center of the hydrogel strand are not provided with enough substrate molecules for maximum velocity. Their contribution to the overall catalytic activity of the system is small and thus, the effectiveness factor decreases.

The numerical solutions of COMSOL in case of sphere and infinite cylinder geometries are in perfect accordance to the analytical solutions, which is why the solutions cannot be distinguished in Figure 2. Only in case of the cross-shaped geometry a slightly higher effectiveness factor is predicted by the analytical solution for a Thiele modulus in the range of ∼1–10. This can be explained by the decreased specific surface of the cross in comparison to the endless plane which is used as approximation in the analytical solution.

**FIGURE 3:**
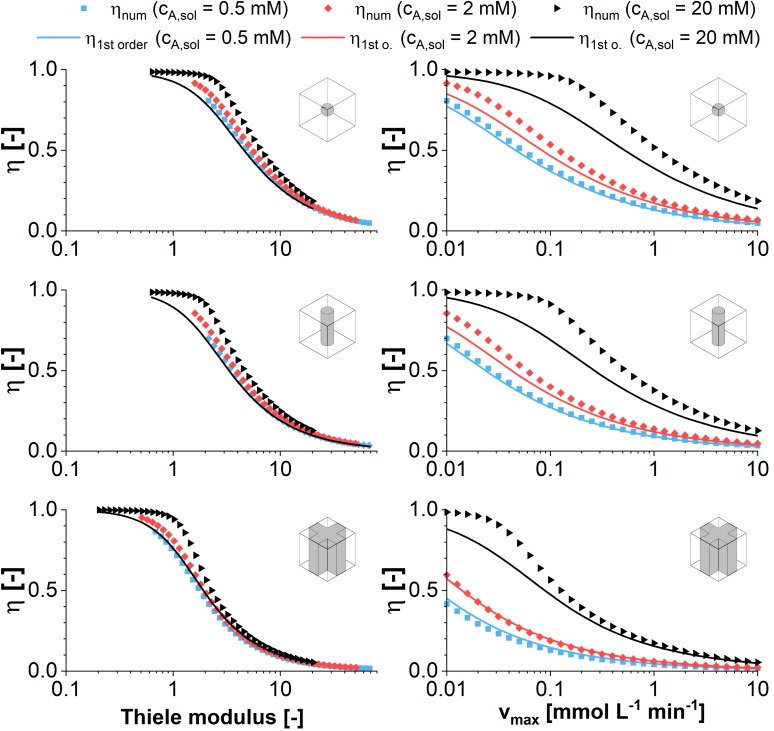
Comparison of analytical (symbols) and numeric (lines) solutions of the effectiveness factor plotted vs. the Thiele modulus and the maximum reaction rate according to Michaelis-Menten kinetics. The effectiveness factor is calculated for the model structures sphere, infinite cylinder, and cross-shaped infinite rod for three exemplary substrate concentrations of 0.5, 2, and 20 mM against the maximum reaction rate. For the analytical solution first order kinetics are assumed using an effective reaction rate constant k_*eff*_ defined in Equation (8). All calculations are performed assuming a diffusion coefficient of 3E–12 m^2^s^–1^ and varying the maximum reaction rate v_*max*_ of the Michealis-Menten kinetics between 0.01and 10 mol m^–3^ s^–1^.

When the Thiele modulus is evaluated for an enzymatic reaction with Michaelis-Menten kinetics and *v*_*max*_ as variable parameter, it is dependent on the substrate concentration in the reaction vessel. As a result, the effectiveness factor differs, too, in a plot against the Thiele modulus (see Figure 3). The differences are highlighted if η is plotted against *v*_*max*_ directly. For high concentrations of *c*_*A,solution*_, the decreasing amount of substrate within the catalyst pellet does not have an immediate effect on the reaction velocity as long as it remains high enough that reaction takes place within the pseudo zero-order kinetics area of the Michaelis-Menten curve. This makes immobilized entities reacting with Michaelis-Menten kinetics more efficient than those reacting with first-order kinetics (Figure 2) in the case of substrate excess. The difference in the effectiveness factor is the more pronounced the higher the ratio between *c*_*A,solution*_ and *K*_*m*_. Analog to first order kinetics, the surface to volume ratio influences the exact curve progression of the effectiveness factor, favoring the sphere geometry if we compare the surface of a sphere with the lateral surface of a cylinder having the same radius^1^. For low substrate concentrations in combination with disadvantageous geometries, the driving forces for the diffusional gradient are weak. As a result, the approximation of an effectiveness factor of one is clearly wrong in this case, because mass transfer limitations dominate the achievable productivity.

Comparing the plots of the effectiveness factor against the Thiele modulus and the maximum reaction velocity *v*_*max*_, it can be stated that the reference to the Thiele modulus is the more useful for a general approach, as the figures approximate a master curve. The visualization of η against *v*_*max*_ is useful in the case of a case study, when optimization potential in case of small changes of *v*_*max*_ are evaluated. Especially for the cross geometry however, the surface to volume ratio will be strongly dependent on the hydrogel strand thicknesses in a unit cell of given dimensions. Having a closer look it shows that there is no global optimum, but an optimum for each enzyme/material combination, which will be discussed in the next section.

### Unit Cell Porosity Optimization

Optimization of the unit cell cannot be done by solving a simple set of non-linear equations, as diffusion, reaction and given geometry interact in a complex way. Therefore, a representative 2D-simulation of a cross-shaped enzyme carrier was established, allowing a fast screening of the effects of geometrical parameters onto the achievable reaction rates by means of a high number of FEM calculations.

**FIGURE 4:**
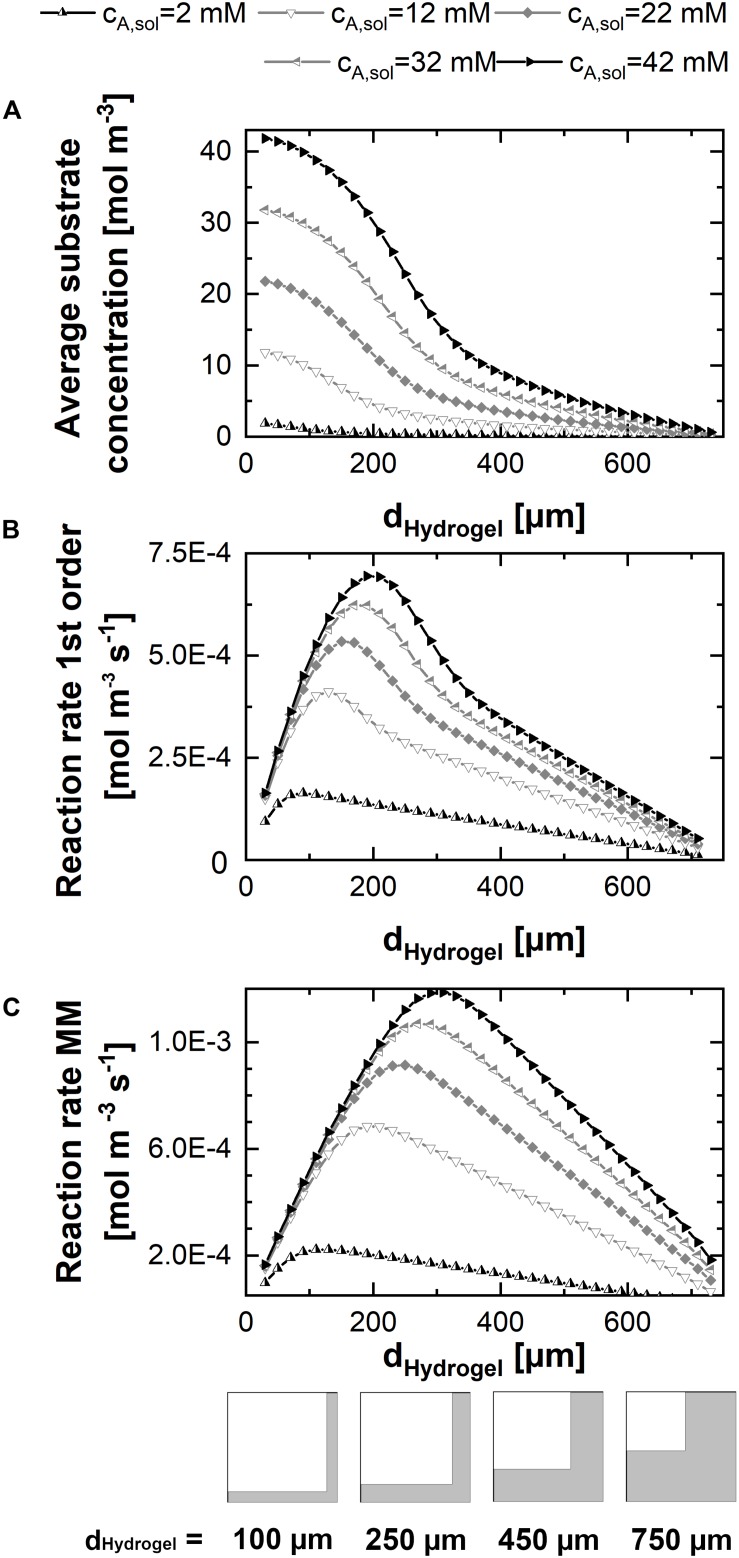
2D-simulations within a unit cell representing a quarter of the cross-shaped hydrogel strand cut-section and the adjacent flow channel, applying varying fractions of hydrogel and water. **(A)** Mean substrate concentration in the hydrogel. **(B)** Productivity within the unit cell in case of pseudo first order kinetics with the reaction rate constant k_*eff*_. **(C)** Productivity within the unit cell in case of Michaelis-Menten kinetics. All calculations were performed assuming a diffusion coefficient of 3E–12 m^2^s^–1^ of the substrate and the product within the hydrogel, five substrate concentrations between 2 and 42 mM, and a unit cell of 0.75 mm edge length.

In order to evaluate the results of a parameter sweep of the hydrogel strand thicknesses in a unit cell of given dimension, at first, the mean substrate concentration within the hydrogel was calculated for different substrate concentrations in the liquid phase at the beginning of the experiment (Figure 4A). Hereby, each marker in the graph represents the stationary solution of a simulation for one parameter set consisting of hydrogel strand width and substrate concentration in the solution. The mean concentration of *c*_*A*_ in the hydrogel is increasing sigmoidal with decreasing hydrogel strand thickness, reaching a plateau value of c_*A,sol*_ in case of strand thicknesses below ∼50 μm. Therefore, at very small hydrogel strand thicknesses, the reaction rate in the hydrogel reaches its maximum. However, this does not mean, that the productivity of the unit cell is high, because the catalytically active hydrogel covers only a small fraction of it.

The contrary effects of decreasing the hydrogel strand thickness, namely increasing the local reaction rate within the hydrogel but on the same time decreasing the fraction of the unit cell which is filled with catalytically active hydrogel are considered in the calculations of the average reaction rates within the unit cell for first order and Michaelis-Menten kinetics (see Figures 4B,C). Both plots have in common that there exists an optimum strand thickness for which the average reaction rate of the unit cell reaches a maximum^2^.

In case of first order reaction kinetics, the reaction rate is linear dependent on the available substrate solution (Figure 4B). For small hydrogel strands the substrate concentration throughout the hydrogel almost equals the concentration in the solution. Therefore, an increase of the average reaction rate within the unit cell is observed which is almost linear to the increasing strand thickness and the corresponding amount of hydrogel per unit cell. When the hydrogel strand thickness is increasing further, the available substrate concentration in the middle of the stand starts to drop, and the added hydrogel results only in a reduced additional contribution to the productivity of the unit cell. Moreover, increased strand thicknesses result in a decrease of the interphase between the hydrogel and the surrounding liquid phase, if the total size of the unit cell is kept constant. This relationship is illustrated in the pictograms at the bottom of Figure 4. Reducing the interphase also reduces the amount of hydrogel which is located in close proximity to the substrate solution and therefore operates at optimum conditions. As a consequence, when increasing the hydrogel strand thickness the average reaction rate of the unit cell shows an optimum. If the hydrogel strand thickness is increased even further, the average reaction rate and therefore the productivity of the unit cell starts to drop, although the amount of hydrogel and therefore the amount of entrapped enzyme within the unit cell increases.

The hydrogel strand thickness that marks the optimum of the calculated reaction rate is dependent on the substrate concentration in the liquid phase (*c_*A*,__*solution*_*). This dependence is even intensified in case of Michelis-Menten kinetics (Figure 4C). When one recalls the typical visualization of the Michaelis-Menten curve, the reaction rate of the product *r*_*B*_ is correlated to *c_*A*,__*solution*_*. At low concentrations of *c_*A*,__*solution*_* there is a pseudo-linear increase of *r*_*B*_,which comes to an end if substrate is available in excess. At this point, the maximum reaction rate is reached and the reaction approaches zero order kinetics. In case of decreased substrate availability in diffusion-controlled environments like the hydrogel lattice in the reactor, this varying slope of *r*_*B*_ has to be considered. Figure 4C shows that in case of Michaelis-Menten kinetics the optimum productivity is reached at a larger hydrogel strand thicknesses than in the case of first order kinetics (Figure 4B). In numbers, for the 12 mM substrate solution, this means a maximum reaction rate of 4.1E–4 mol m^–3^s^–1^ for first order kinetics and hydrogels of 130 μm thickness. The same unit cell structure is able to reach a reaction rate of 5.8E–4 mol m^–3^s^–1^ for Michaelis-Menten kinetics, which is an increase of 40%. And what’s more, applying a substrate concentration of 12 mM and Michaelis-Menten kinetics results in the maximum reaction rate of 6.8E–4 mol m^–3^s^–1^, corresponding to a 67% increase, calculated for 190 μm hydrogel strands. On top of that, the overall reaction rate decreases only linear when the hydrogel strand thickness is increased further, in comparison to the approximately exponential decrease in case of first order kinetics.

While this parameter sweep calculated in a 2D system clearly shows the existence of an optimum and the influence of the strand thickness as well as of the substrate concentration in the solution, it cannot predict how the substrate and product concentrations will vary along the flow path within the enzyme reactor. Therefore, the investigated geometry was extended to a 3D unit cell.

### Conversion in the 3D-System

**FIGURE 5:**
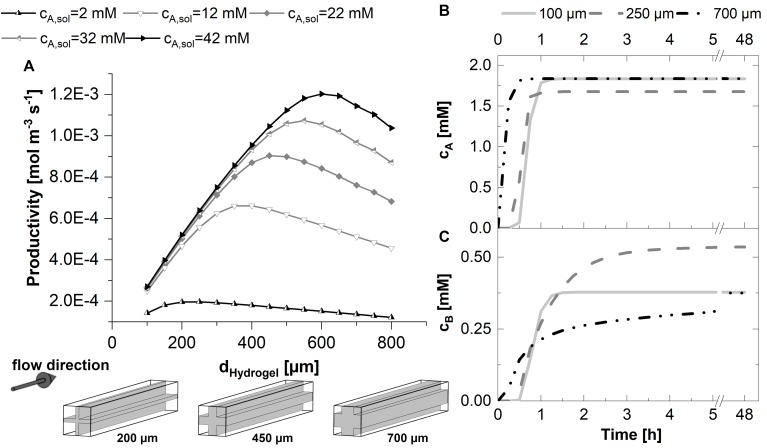
3D-simulations within a flow unit cell representing the cross-shaped hydrogel strand and the adjacent flow channels. Michaelis-Menten kinetics are assumed in all cases. **(A)** Productivity within the 3D-unit cell, assuming five different substrate concentrations in the inlet between 2 and 42 mM, and hydrogel strand thicknesses between 100 and 750 μm. The small graphics at the bottom of plot A illustrate the ratio between the hydrogel and flow channels in dependence of the thickness d_*hydrogel*_ of the hydrogel strand. **(B,C)** Time-dependent concentration profiles in the effluent of the 3D-unit cell for substrate **(B)** and product **(C)**. In the calculations associated with **(B,C)**, the substrate concentration in the inlet was set to 2.2 mM. All calculations were performed assuming a diffusion coefficient of 3E-12 m^2^s^–1^ of the substrate and the product within the hydrogel, a unit cell cross-section of 1.5 mm edge length and a cell length of 15 mm.

The productivity of the 3D unit cell was calculated and depicted in Figure 5A. As in the case of the 2D model, the productivity of the 3D unit cell increases nearly linear with increasing strand thicknesses if the strands are in the range of 100–200 μm in a 1.5 mm unit cell. The optimum strand thickness is again dependent on the substrate concentration, starting from 200 μm strands for 2 mM substrate solution to 600 μm for 42 mM solution, where substrate excess is very high.

For the time-dependent yield of the process and the estimation of the duration of the start-up phase, the product concentration in the solution at the exit of the unit cell is relevant. At the beginning of the experiment, the reactor chamber is filled with buffer. While buffer is replaced by substrate solution, the product formation within the system rises sharply. The duration of this initial phase until a stationary conversion at the exit of the reactor is reached depends on the hydrogel strand thickness and thus the mass transfer limitation as well as the accumulation capacity within the hydrogel. Figures 5B,C show the time course of the effluent concentration of the substrate as well as the product for three exemplary hydrogel strand thicknesses. In case of the exemplary diffusion-limited system, the substrate is completely consumed in the outer rim of the hydrogel strands. Therefore, stationary concentrations of the substrate at the exit of the reactor are reached in a short time. In contrast, the product spreads within the full hydrogel phase according to local concentration gradients. As a result, with respect to the product concentration in the effluent, an exponential elongation of the initial phase of the reactor performance is expected for increasing hydrogel strand thicknesses.

**FIGURE 6:**
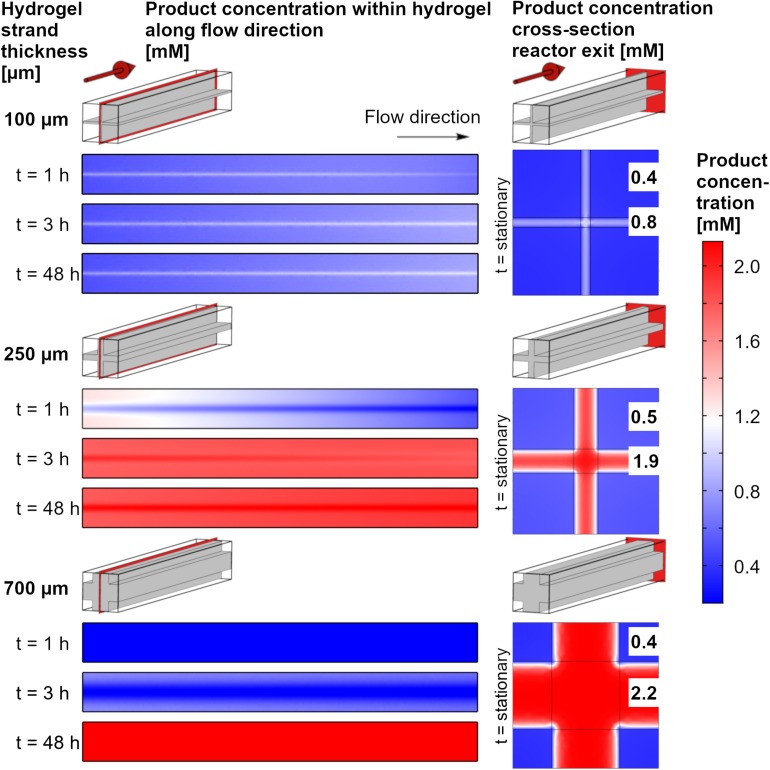
Color code maps for local product concentrations at the central axial cross-section of the reactor and the cross section of the reactor exit. The data are shown for a substrate concentration of 2.2 mM, three exemplary hydrogel strand thicknesses in the 1.5 mm unit cell and three operating times of the system. The numbers in the plots at the right side indicate the stationary values (48 h) of the product concentration in the center of the flow channel and in the center of the hydrogel slabs.

The product accumulation can be visualized by the local concentrations of the product within the reactor over time (Figure 6). The cross-sections of the reactor unit cells along the flow direction show the center of the hydrogel strand. Local product concentrations vary over the length of the system, but the concentration gradients between the rim of the hydrogel strand and its central part is even more pronounced for all time points and hydrogel strand widths. This can be discussed exemplarily for the unit cell with a cross of two 250 μm hydrogel strands. After 1 h, which is in the start-up phase, the product concentration is highest within the hydrogel parts which are close to the inlet of the reactor. In addition, because of the easier access of the substrate the flat plate like regions between the strand crossings shows higher product concentrations than the region at the center of the crossing. The local concentration in the flat regions of the hydrogel strands at the inlet approaches 1.2 mM, whereas at the center of the crossings in the inlet regions the local product concentration is only about 0.8 mM. As the product profile shows decreasing concentrations toward the strand crossing, it can be assumed that part of the local product is not a result of local enzymatic conversion, but of product diffusion within the hydrogel. After 3 h of reaction, steady state is almost reached (see also Figure 5C), and the product profile over the length of the reactor is almost constant over the whole reactor chamber length. After 48 h the reactor reached its steady state, and the product concentrations within the hydrogel are highest in the center at the crossings of the strands. This means at some point of the process, the direction of product diffusion within the hydrogel is inverted.

For 100 μm strands, steady state will adjust itself very quickly because of the short diffusion distance and the low accumulation of product. Economically, this is the process with the best use of the immobilized enzymes. The overall productivity of the system, however is small, as most of the substrate solution will pass the hydrogel unreacted. For the 700 μm strands, accumulation of the product will prolong the start-up phase. If the operation time of the system is increased, this accumulated product amount gets neglectable.

**FIGURE 7:**
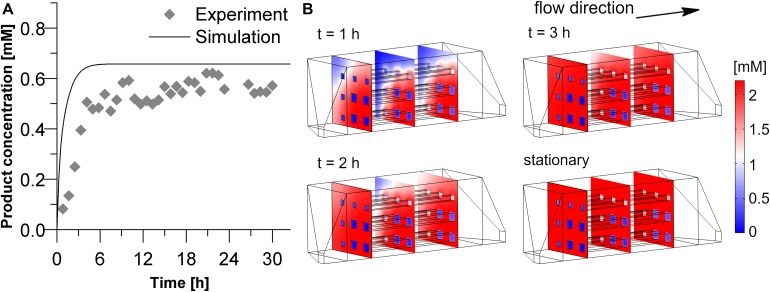
Product concentration in the inner volume as well as in the effluent solution of the experimental reactor (V_*chamber*_ = 3 mL, hydrogel strand thickness 700 μm with thicker strands at the outer regions due to printer settings). **(A)** Comparison of the experimental and simulated data of the product concentration in the effluent. **(B)** Product concentration profiles within the reactor chamber during the starting phase of the reaction and the stationary state. The calculation was performed with a substrate concentration in the inlet of 2.2 mM, and a diffusion coefficient of 3E–12 m^2^s^–1^ of the substrate and the product within the hydrogel.

As a final step, the geometry of the hydrogel structures used in our experiments was implemented in the 3D simulation and the predicted results were compared with the experimental data (see Figure 7). The simulation predicts a relatively fast increase of product exiting the reactor. Three hours after the start of the reaction, the stationary state of 0.66 mM product is nearly reached. In the experiment, however, about 6 h passed to approach stationary yield. In the stationary phase, product concentration equaled 0.56 mM. A quantitative comparison between the experimental and simulated product concentration in the stationary phase shows that the model overestimates the product formation by 0.1 mM. If this deviation is normalized by the maximum product concentration achievable in this experiment (2.2 mM) it reveals that the deviation is only around 5%. Another way of normalization can be done by using the average experimental value in the stationary phase as 100%. In this case the relative error of the predicted product concentration in the effluent would be around 18%. Deviations between simulation and experiment may result from the assumption that the intrinsic activity of the entrapped enzyme equals exactly the one of the free enzyme and from non-idealities in the printed channel structure. In case of the delayed initial rise of the product concentration the observed adsorption of reactants to the 3D-printed parts of the system may play a role, an effect which was disregarded in the simulative setup.

## Conclusion

3D-printing offers new possibilities for the geometric design of enzyme carriers. For continuous reactions in fixed-bed reactors, the porosity and structure dimensions of the fixed bed can be chosen in a wide range and replace the commonly used particle bed. In contrast to monolithic structures that are produced *in situ*, modular production enables flexibility and scalability.

The performance of the entrapped enzymes – apart from the individual activity loss of the catalyst by immobilization – depends on the equilibrium between reaction rate and mass transfer within the bioprinted hydrogels. The availability of high amounts of substrate at the site of reaction will hereby positively influence the overall process performance, as many enzymes react according to Michaelis-Menten kinetics. In the case of substrate excess, the reaction rate approaches zero order kinetics, which means a that the productivity within the hydrogel stays close to its maximum although the substrate concentration decreases because of mass transfer limitations.

Experimentally however, most reactions are conducted under substrate-limited conditions and diffusion-controlled kinetics. A simulative variation of the geometry of the enzyme carrier helps to visualize areas of high mass transfer limitations. Moreover, the optimization potential within the range of the feasible range of the printing resolution can be estimated.

In this article, each simulation approach emphasizes one of the factors influencing mass transfer limitation. While the Thiele modulus and the effectiveness factor describe the overall process performance, the 2D simulation highlights the existence of an optimum strand thickness representing the best compromise between large interphase areas favored by thin strands and large enzymatically active hydrogel volumes favored by thick strands. Finally, the full 3D simulation showed, that it is possible to predict the productivity of the reactor system applying 3D-printed enzyme immobilizates, if the kinetics of the free enzyme and the diffusivity of the substrate in the hydrogel are known. In addition to the local substrate gradients within the hydrogel described in 2D, the 3D simulation also takes into account the substrate gradients along the flow path within the reactor. Besides stationary solutions, the simulation also predicts the duration of the initial phase of the reactor performance, which is mainly dominated by the accumulation of the product within the hydrogel.

In early stage process development, the modular simulative approach can be used for the design of a tailor-made geometry adapted to the individual enzyme reaction kinetics. Production by 3D-printing as a quasi-monolithic building block or in a modular setup with varying strand thicknesses and channel widths enables fast testing with a minimum of material consumption.

## Author Contributions

BS executed the simulations and wrote the manuscript together with MF, who developed the concept of the paper. MN conducted preliminary studies under the supervision of MF and BS.

## Conflict of Interest

The authors declare that the research was conducted in the absence of any commercial or financial relationships that could be construed as a potential conflict of interest.

## Footnotes

1 The well-known fact that a sphere is the geometry having the smallest volume specific surface does not contradict this statement, because the simulated model geometries do not have the same volume and the front surfaces of the cylinder and the cross do not contribute in this comparison.

2 It should be mentioned that the calculation assumes that only one horizontally and one vertically oriented strand can be printed within the area of a unit cell. This assumption is justified because of the limited resolution of extrusion-based 3D-printers. Printing strands in too close proximity implies the risk that the hydrogels merge and an undefined geometrical structure is formed.
