# Supplementary material for: Simulative Minimization of Mass Transfer Limitations Within Hydrogel-Based 3D-Printed Enzyme Carriers
Source: Front Bioeng Biotechnol. 2020 Apr 28;8:365. doi: 10.3389/fbioe.2020.00365 (PMC7198751; doi:10.3389/fbioe.2020.00365)

1

Simulation Report 3D Unit Cell

2 Global Definitions

GLOBAL SETTINGS

Name	unit cell 3D.mph
Version	COMSOL Multiphysics 5.4 (Build: 346)

USED PRODUCTS

COMSOL Multiphysics
Batteries & Fuel Cells Module
CAD Import Module

2.1 PARAMETERS

PARAMETER 1

Name	Expression	Value	Description
Phi	((1/2)*d_Strang)*sqrt(k_eff/D_HG)	4.9485	Thiele Modulus
u_Water	(1/256)*V_dot/A_Waterchannel	2.0345E−5 m/s	av. flow velocity within the channel
A_Waterchannel	(d_Zelle-0.5*d_Strang)^2	1.6E−7 m^2	cross section fluid channel
vmax	0.13 [mmol/(L*min)]	0.0021667 mol/(m^3·s)	max. reaction rate
d_Cell	0.75 [mm]	7.5E−4 m	size unit cell
d_Strand	0.7E-3 [m]	7E−4 m	size hydrogel strand
Km	1.4 [mmol/L]	1.4 mol/m^3	Km-value Michaelis-Menten
L_Strand	15 [mm]	0.015 m	length of the 3D unit cell
c_A_bulk	2.213[mol/m^3]	2.213 mol/m^3	inlet concentration
V_dot	3 [ml/h]	8.3333E−10 m^3/s	volumetric flow
D_HG	3E-12[m^2/s]	3E−12 m^2/s	eff. diffusivity with the hydrogel
V_cross	L_Strand*(d_Cell^2 - A_Waterchannel)	6.0375E−9 m^3	volume hydrogel
eta	tanh(Phi)/Phi	0.20206	effectiveness factor
k_eff	vmax/(Km + c_A_bulk)	5.9969E−4 1/s	apparent reaction rate 1.st order

3 Hydrogel Channel

SETTINGS

Description	Value
Unit system	Same as global system

3.1 GEOMETRY

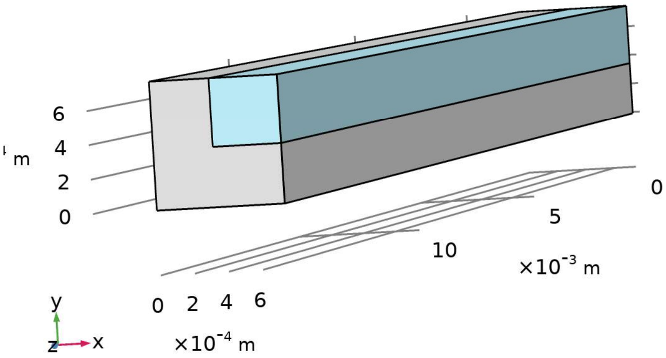

Geometry

UNITS

Length unit	m
Angular unit	deg

GEOMETRY STATISTICS

Description	Value
Space dimension	3
Number of domains	2
Number of boundaries	12
Number of edges	23
Number of vertices	14

3.1.1 Cuboid 1 (blk1)

POSITION

Description	Value
Position	{0, 0, 0}

AXIS

Description	Value
Axis type	z - axis

SIZE AND SHAPE

Description	Value
Width	d_Cell
Depth	d_Cell
Height	L_Strand

3.1.2 Cuboid 2 (blk2)

POSITION

Description	Value
Position	{d_Strand/2, d_Strand/2, 0}

AXIS

Description	Value
Axis type	z - axis

SIZE AND SHAPE

Description	Value
Width	d_Cell-d_Strand/2
Depth	d_Cell-d_Strand/2
Height	L_Strand

3.1.3 Difference 1 (dif1)

SELECTIONS OF RESULTING ENTITIES

Description	Value
Contribute to	Hydrogel
Resulting objects selection	On
Color	Custom
Custom color	{0.8627451062202454, 0.8705882430076599, 0.8705882430076599}

3.1.4 Cuboid 3 (blk3)

SELECTIONS OF RESULTING ENTITIES

Description	Value
Contribute to	Water

Description	Value
Resulting objects selection	On
Color	Custom
Custom color	{0.7254902124404907, 0.9215686321258545, 0.9686274528503418}

POSITION

Description	Value
Position	{d_Strand/2, d_Strand/2, 0}

AXIS

Description	Value
Axis type	z - axis

SIZE AND SHAPE

Description	Value
Width	d_Cell-d_Strand/2
Depth	d_Cell-d_Strand/2
Height	L_Strand

3.2 TRANSPORT OF DILUTED SPECIES IN POROUS MEDIA

USED PRODUCTS

COMSOL Multiphysics
Batteries & Fuel Cells Module

EQUATIONS

$$\nabla \cdot \mathbf{J}_i + \mathbf{u} \cdot \nabla c_i = R_i + S_i$$
$$\mathbf{J}_i = -D_{\mathrm{e},i} \nabla c_i$$
$$\theta = \epsilon_{\mathrm{p}}$$

3.2.1 Hydrogel Transport Properties

EQUATIONS

$$\nabla \cdot \mathbf{J}_i + \mathbf{u} \cdot \nabla c_i = R_i + S_i$$
$$\mathbf{J}_i = -D_{\mathrm{e},i} \nabla c_i$$
$$\theta = \epsilon_{\mathrm{p}}$$

Matrix properties

SETTINGS

Description	Value
Porous material	Domain material
Porosity	From material

Convection

SETTINGS

Description	Value
Velocity field	User defined
Velocity field	{0, 0, 0}

Diffusion

SETTINGS

Description	Value
Fluid material	Hydrogel (mat1)
Fluid diffusion coefficient	User defined
Fluid diffusion coefficient	{{D_HG, 0, 0}, {0, D_HG, 0}, {0, 0, D_HG}}
Fluid diffusion coefficient	User defined
Fluid diffusion coefficient	{{D_HG, 0, 0}, {0, D_HG, 0}, {0, 0, D_HG}}
Effective diffusivity model	No correction

Coordinate system selection

SETTINGS

Description	Value
Coordinate system	Global coordinate system

Model input

SETTINGS

Description	Value
Temperature	User defined
Temperature	293.15[K]

PROPERTIES FROM MATERIAL

Property	Material	Property group
Porosity	Hydrogel	basic

3.2.2 No Flux

EQUATIONS

$$-\mathbf{n} \cdot \mathbf{J}_i = 0$$

Convection

SETTINGS

Description	Value
Include	Off

3.2.3 Initial Values

Initial values

SETTINGS

Description	Value
Concentration	{0, 0}

3.2.4 Reactions

EQUATIONS

$$\nabla \cdot \mathbf{J}_i + \mathbf{u} \cdot \nabla c_i = R_i + S_i$$

Reaction rates

SETTINGS

Description	Value
Total rate expression	User defined
Total rate expression	-((vmax*c_A)/(Km + c_A))
Total rate expression	User defined
Total rate expression	+((vmax*c_A)/(Km + c_A))

Reacting volume

SETTINGS

Description	Value
Reacting volume	Total volume

3.2.5 Water Transport Properties

EQUATIONS

$$\nabla \cdot \mathbf{J}_i + \mathbf{u} \cdot \nabla c_i = R_i$$

$$\mathbf{J}_i = -D_i \nabla c_i$$

Convection

SETTINGS

Description	Value
Velocity field	User defined

Description	Value
Velocity field	{0, 0, u_Water}

Diffusion

SETTINGS

Description	Value
Material	Water (mat2)
Diffusion coefficient	User defined
Diffusion coefficient	{{1e-9[m^2/s], 0, 0}, {0, 1e-9[m^2/s], 0}, {0, 0, 1e-9[m^2/s]}}
Diffusion coefficient	User defined
Diffusion coefficient	{{1e-9[m^2/s], 0, 0}, {0, 1e-9[m^2/s], 0}, {0, 0, 1e-9[m^2/s]}}

Coordinate system selection

SETTINGS

Description	Value
Coordinate system	Global coordinate system

Model input

SETTINGS

Description	Value
Temperature	User defined
Temperature	293.15[K]

3.2.6 Symmetry

EQUATIONS

$$-\mathbf{n}\cdot\mathbf{J}_i=0$$

Convection

SETTINGS

Description	Value
Include	Off

3.2.7 Inflow

EQUATIONS

$$\mathbf{n}\cdot(\mathbf{J}_i+\mathbf{u}c_i)=\mathbf{n}\cdot(\mathbf{u}c_{0,j})$$

Concentration

SETTINGS

Description	Value
Concentration	{c_A_bulk, 0}

Boundary condition type

SETTINGS

Description	Value
Boundary condition type	Flux (Danckwerts)

3.2.8 Outflow

EQUATIONS

$$\mathbf{n} \cdot D_i \nabla c_i = 0$$

3.3 MESH

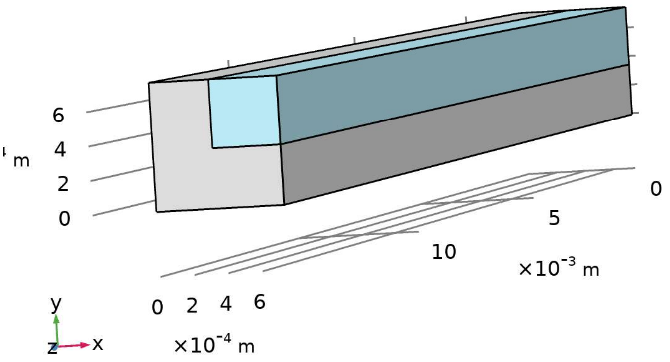

Mesh

3.3.1 Size (size)

SETTINGS

Description	Value
Maximum element size	0.0015
Minimum element size	2.7E-4
Curvature factor	0.6
Resolution of narrow regions	0.5
Maximum element growth rate	1.5

3.3.2 Free Tetrahedral (ftet1)

SELECTION

Geometric entity level	Domain
Selection	Remaining

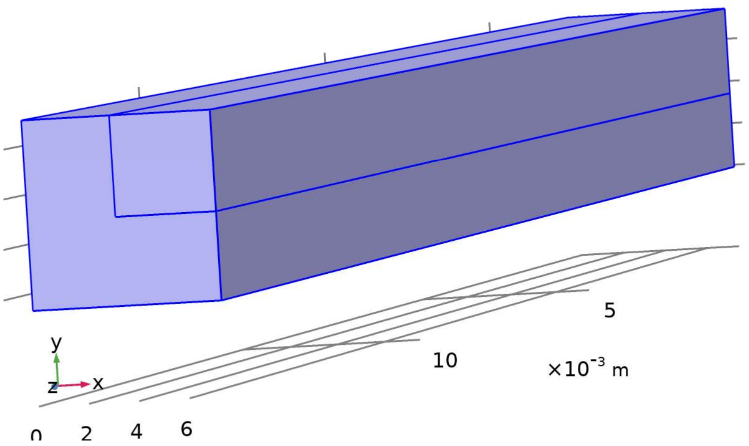

Free Tetrahedral

SETTINGS

Description	Value
z-direction scale	0.2

3.3.3 Boundary Layers (bl1)

SELECTION

Geometric entity level	Domain
Selection	Domain 1

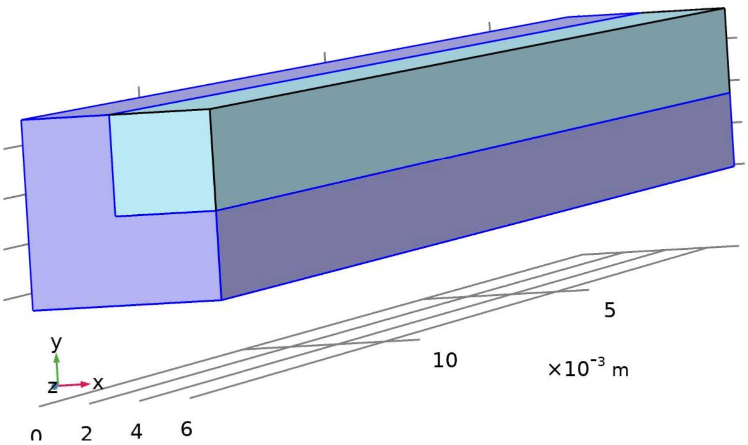

Boundary Layers

Properties of the Boundary Layers (blp)

SELECTION

Geometric entity level	Boundary
Selection	Boundaries 6–7

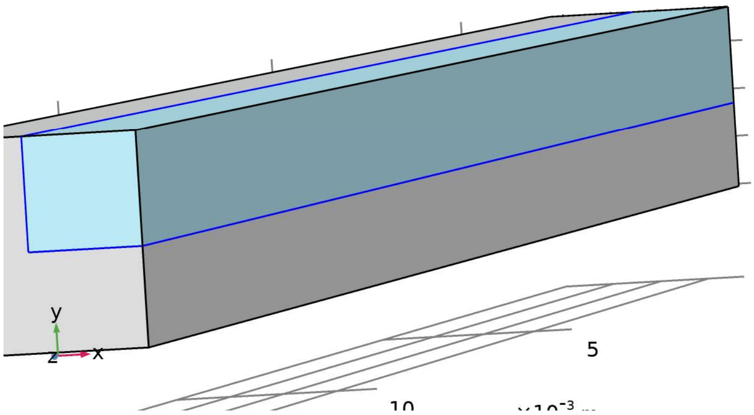

Properties of the Boundary Layers

4 Stationary

COMPUTATION INFORMATION

Computation time	1 min 39 s

CPU	Intel64 Family 6 Model 142 Stepping 9, 4 cores
Operating system	Windows 10

4.1 SOLVER CONFIGURATIONS

4.1.1 Solution 1

Compile Equations: Stationary (st1)

STUDY AND STEP

Description	Value
Use study	Stationary
Use study step	Stationary

Dependent Variables 1 (v1)

GENERAL

Description	Value
Defined by study step	Stationary

INITIAL VALUE CALCULATION CONSTANTS

Constant name	Initial value source
c_A_bulk	range(2,10,42)[mol/m^3]

Concentration (comp1.c_A) (comp1_c_A)

GENERAL

Description	Value
Field components	comp1.c_A

Concentration (comp1.c_B) (comp1_c_B)

GENERAL

Description	Value
Field components	comp1.c_B

Stationary Solver 1 (s1)

GENERAL

Description	Value
Defined by study step	Stationary

RESULTS WHILE SOLVING

Description	Value
Probes	None

Parametric 1 (p1)

PARAMETERS

Parameter name	Parameter value list	Parameter unit
c_A_bulk	range(2,10,42)	mol/m^3

Fully coupled 1 (fc1)

GENERAL

Description	Value
Linear solver	Iterativ 1

METHOD AND TERMINATION

Description	Value
Initial damping factor	0.01
Minimum damping factor	1.0E-6
Maximum number of iterations	50

Iterative 1 (i1)

GENERAL

Description	Value
Nonlinear-based error norm	On
Maximum number of iterations	400

ERROR

Description	Value
Factor in error estimate	40

Multigrid 1 (mg1)

GENERAL

Description	Value
Use hierarchy in geometries	Geometry

Presmoother (pr)

SOR Line 1 (sl1)

MAIN

Description	Value
Relaxation factor	0.2

SECONDARY

Description	Value
Relaxation factor	0.4

Postsmoother (po)

SOR Line 1 (sl1)

MAIN

Description	Value
Relaxation factor	0.2

SECONDARY

Description	Value
Number of secondary iterations	2
Relaxation factor	0.4

Coarse Solver (cs)

Direct 1 (d1)

GENERAL

Description	Value
Solver	PARDISO
Pivoting perturbation	1.0E-13

5 Dynamic

COMPUTATION INFORMATION

Computation time	12 s
CPU	Intel64 Family 6 Model 142 Stepping 9, 4 cores
Operating system	Windows 10

5.1 SOLVER CONFIGURATIONS

5.1.1 Solution 2

Compile Equations: Dynamic (st1)

STUDY AND STEP

Description	Value
Use study	Dynamic
Use study step	Dynamic

Dependent Variables 1 (v1)

GENERAL

Description	Value
Defined by study step	Dynamic

RESIDUAL SCALING

Description	Value
Method	Manual

INITIAL VALUE CALCULATION CONSTANTS

Constant name	Initial value source
t	range(0,0.25,24)
timestep	0.024[h]

Concentration (comp1.c_A) (comp1_c_A)

GENERAL

Description	Value
Field components	comp1.c_A

Concentration (comp1.c_B) (comp1_c_B)

GENERAL

Description	Value
Field components	comp1.c_B

Time-dependent Solver 1 (t1)

GENERAL

Description	Value
Times	{0, 0.25, 0.5, 0.75, 1, 1.25, 1.5, 1.75, 2, 2.25, 2.5, 2.75, 3, 3.25, 3.5, 3.75, 4, 4.25, 4.5, 4.75, 5, 5.25, 5.5, 5.75, 6, 6.25, 6.5, 6.75, 7, 7.25, 7.5, 7.75, 8, 8.25, 8.5, 8.75, 9, 9.25, 9.5, 9.75, 10, 10.25, 10.5, 10.75, 11, 11.25, 11.5, 11.75, 12, 12.25, 12.5, 12.75, 13, 13.25, 13.5, 13.75, 14, 14.25, 14.5, 14.75, 15, 15.25, 15.5, 15.75, 16, 16.25, 16.5, 16.75, 17, 17.25, 17.5, 17.75, 18, 18.25, 18.5, 18.75, 19, 19.25, 19.5, 19.75, 20, 20.25, 20.5, 20.75, 21, 21.25, 21.5, 21.75, 22, 22.25, 22.5, 22.75, 23, 23.25, 23.5, 23.75, 24}
Relative tolerance	0.005

TIME STEPPING

Description	Value
Maximum BDF order	2

Fully coupled 1 (fc1)

GENERAL

Description	Value
Linear solver	Iterative 1

METHOD AND TERMINATION

Description	Value
Damping factor	0.9
Jacobian update	Once per time step
Maximum number of iterations	8
Stabilization and acceleration	Anderson acceleration
Dimension of iteration space	5
Mixing parameter	0.9
Iteration delay	1

Iterative 1 (i1)

GENERAL

Description	Value
Maximum number of iterations	400

ERROR

Description	Value
Factor in error estimate	40

Multigrid 1 (mg1)

GENERAL

Description	Value
Use hierarchy in geometries	Geometry

Presmoother(pr)

SOR Line 1 (sl1)

MAIN

Description	Value
Relaxation factor	0.2

SECONDARY

Description	Value
Relaxation factor	0.4

Postsmoother (po)

SOR Line 1 (sl1)

MAIN

Description	Value
Relaxation factor	0.2

SECONDARY

Description	Value
Number of secondary iterations	2
Relaxation factor	0.4

Coarse Solver (cs)

Direct 1 (d1)

GENERAL

Description	Value
Solver	PARDISO
Pivoting perturbation	1.0E-13

6 Results

6.1 DERIVED PARAMETERS

6.1.1 Flow channel center Stationary Parametric

DATA

Description	Value
Data set	Stationary Parametric Results

EXPRESSIONS

Expression	Unit	Description
c_A	mol/m^3	Concentration

6.1.2 Flow channel center Dynamic

OUTPUT

Evaluated in	Dynamic concentration profile in the effluent

DATA

Description	Value
Data set	Dynamic/Lösung 2

EXPRESSIONS

Expression	Unit	Description
c_B	mol/m^3	Concentration of product
c_A	mol/m^3	Concentration of substrate

6.2 PLOT GROUPS

6.2.1 Dynamic concentration profile in the effluent

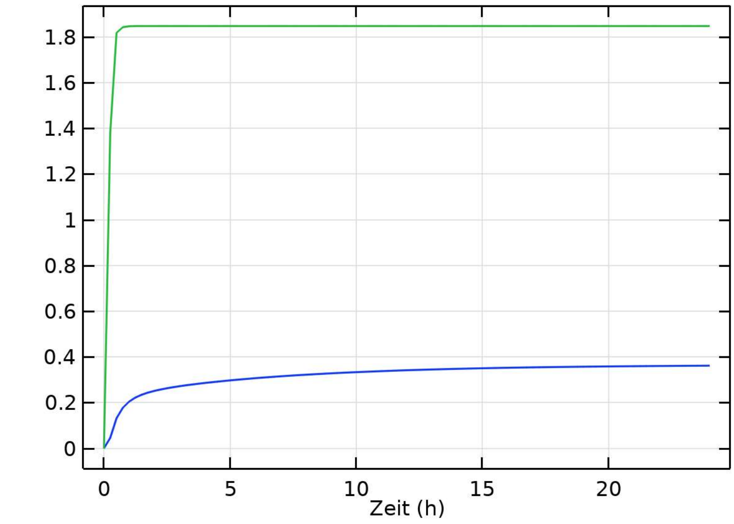

6.2.2 Productivity per unit cell

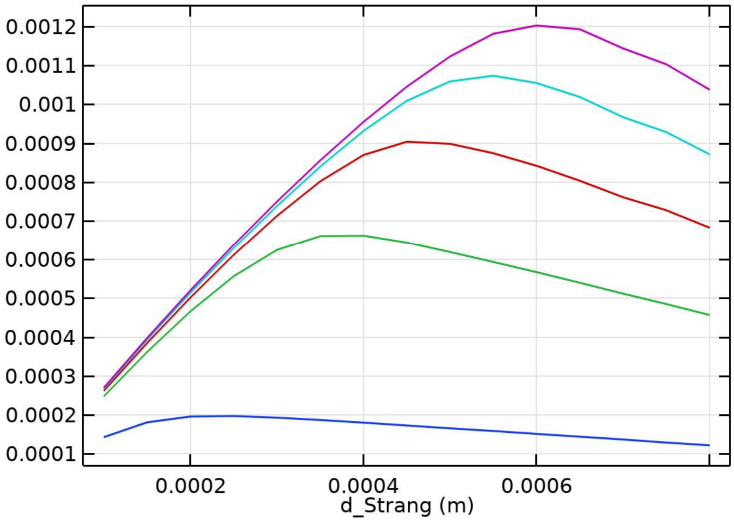

Supplement: Supplementary file 1 [file Data_Sheet_1.PDF]
