# Supplementary material for: Simulative Minimization of Mass Transfer Limitations Within Hydrogel-Based 3D-Printed Enzyme Carriers
Source: Front Bioeng Biotechnol. 2020 Apr 28;8:365. doi: 10.3389/fbioe.2020.00365 (PMC7198751; doi:10.3389/fbioe.2020.00365)

1

Simulation report unit cell 2D

Report date

2 Global Definitions

GLOBAL SETTINGS

Name	unit cell 2D.mph
Version	COMSOL Multiphysics 5.4 (Build: 346)

USED PRODUCTS

COMSOL Multiphysics
Batteries & Fuel Cells Module

2.1 PARAMETERS

PARAMETERS 1

Name	Expression	Value	Description
c_A_bulk	20 [mol/m^3]	20 mol/m³	bulk concentration
D_HG	3E-12[m^2/s]	3E−12 m²/s	
d_Strand	200[µm]	2E−4 m	size hydrogel strand
eta	3/Phi*(1/tanh(Phi) - 1/Phi)	0.9782	effectiveness factor
k_eff	vmax/(Km + c_A_bulk)	1.0125E−4 1/s	Kinetic parameter; see equation (8) in manuscript
Km	1.4[mmol/L]	1.4 mol/m³	Km-value Michaelis-Menten
Phi	((1/2)*d_Strand)*sqrt(k_eff/D_HG)	0.58094	Thiele Modulus
A_Cross	d_Strand^2/4	1E−8 m²	Cross section
vmax	0.13 [mmol/(L*min)]	0.0021667 mol/(m³·s)	vmax-value Michaelis-Menten
kLA	1E-5 [m/s]	1E−5 m/s	mass transport coefficient
a_cell	750[µm]	7.5E−4 m	length of unit cell
b_cell	300[µm]	3E−4 m	length of open channel
V_ges	a_cell*a_cell	5.625E−7 m²	total area unit cell

3 Component 1

3.1 DEFINITIONS

3.1.1 Coordinate Systems

Boundary System 1

Coordinate system type	Boundary system
Tag	sys1

COORDINATE NAMES

First	Second	Third
t1	n	to

3.2 GEOMETRY

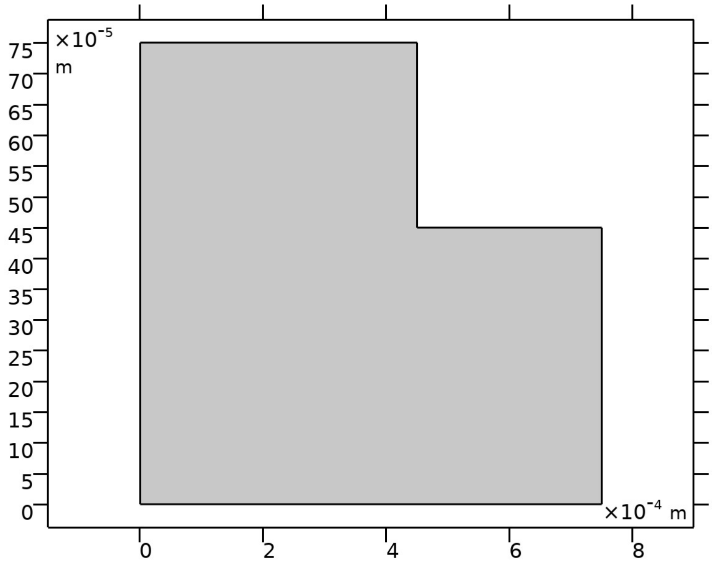

Geometry

UNITS

Length unit	m
Angular unit	deg

3.3 COUPLED DIFFUSION AND REACTION

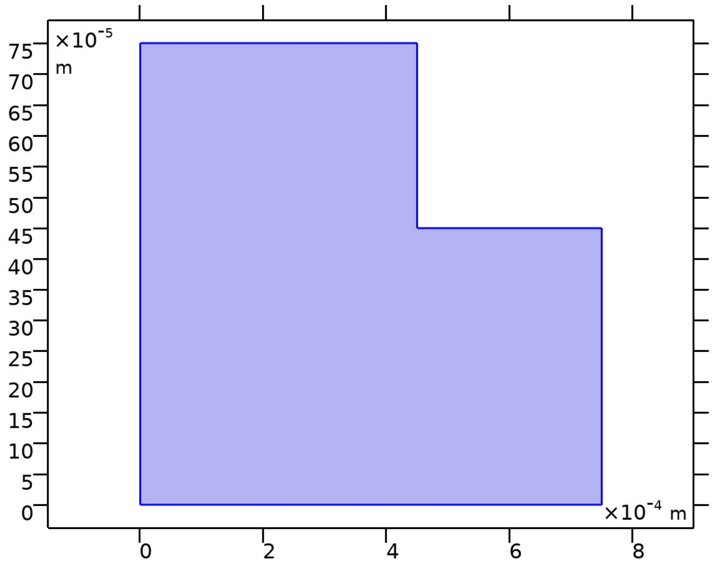

Coupled Diffusion and Reaction

EQUATIONS

$$\mathbf{J}_i = -D_{e,i}\nabla c_i$$
$$\theta = \epsilon_p$$

FEATURES

Porous Media Transport Properties
No Flux
Inital Values
Reactions
Concentration

3.3.1 Porous Media Transport Properties

EQUATIONS

$$\mathbf{J}_i = -D_{e,i}\nabla c_i$$
$$\theta = \epsilon_p$$

3.3.2 No Flux

EQUATIONS

$$-\mathbf{n}\cdot(\mathbf{J}_i + \mathbf{u}c_i) = 0$$

3.3.3 Reactions

EQUATIONS

$$\frac{\partial(\theta c_i)}{\partial t}+\frac{\partial(\rho c_{P,i})}{\partial t}+\nabla\cdot\mathbf{J}_i=R_i+S_i$$

3.3.4 Concentration

EQUATIONS

$$c_i=c_{0,i}$$

3.4 MESH

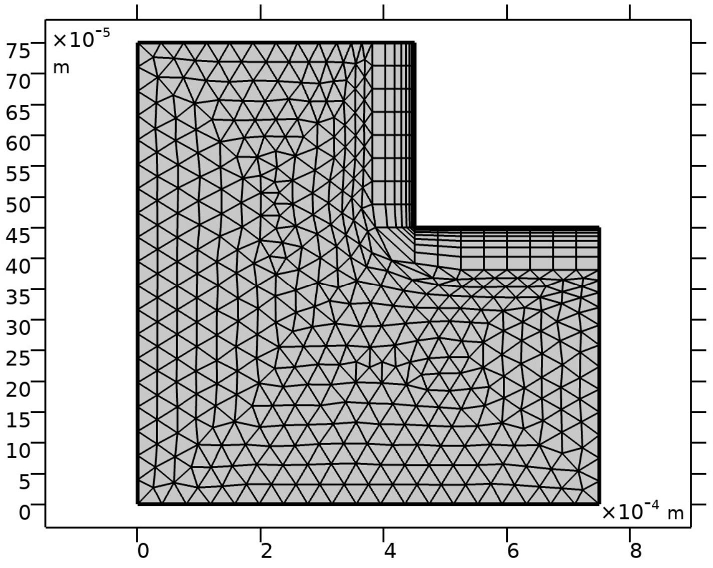

Mesh

4 Study 2

COMPUTATION INFORMATION

Computation time	38 s
CPU	Intel64 Family 6 Model 142 Stepping 9, 4 cores
Operating system	Windows 10

4.1 PARAMETRIC SWEEP

Parameter name	Parameter value list	Parameter unit
b_cell	range(20,20,730)	µm

STUDY SETTINGS

Description	Value
Sweep type	Specified combinations
Parameter name	b_cell
Unit	µm

PARAMETERS

Parameter name	Parameter value list	Parameter unit
b_cell (length of open channel)	range(20,20,730)	µm

4.2 STATIONARY

STUDY SETTINGS

Description	Value
Include geometric nonlinearity	Off

MESH SELECTION

Geometry	Mesh
mesh1	mesh1

PHYSICS AND VARIABLES SELECTION

Physics interface	Discretization
Coupled Diffusion and Reaction (tds)	physics

MESH SELECTION

Geometry	Mesh
Geometry (geom1)	mesh1

4.2.1 Study extensions

STUDY EXTENSIONS

Description	Value
Sweep type	Specified combinations

PARAMETERS

Parameter name	Parameter value list	Parameter unit
c_A_bulk (bulk concentration)	range(2,10,42)	mol/m^3

5 Results

5.1 DERIVED PARAMETERS

5.1.1 average substrate concentration within hydrogel

OUTPUT

Evaluated in	average substrate concentration within hydrogel

DATA

Description	Value
Data set	Parametric Sweep

EXPRESSIONS

Expression	Unit	Description
c_A	mol/m^3	Concentration of substrate

INTEGRATION SETTINGS

Description	Value
Integration order	4

5.1.2 productivity apparent 1st order

OUTPUT

Evaluated in	productivity apparent 1st order

DATA

Description	Value
Data set	Parametric Sweep

EXPRESSIONS

Expression	Unit	Description
c_A*k_eff/(a_cell*a_cell)	mol/(m^3*s)	

INTEGRATION SETTINGS

Description	Value
Integration order	4

5.1.3 productivity MM

OUTPUT

Evaluated in	productivity MM

DATA

Description	Value
Data set	Parametric Sweep

EXPRESSIONS

Expression	Unit	Description
((vmax*c_A)/(Km+c_A))/(a_cell*a_cell)	mol/(m^3*s)	

INTEGRATION SETTINGS

Description	Value
Integration order	4

5.2 PLOT GROUPS

5.2.1 Concentration

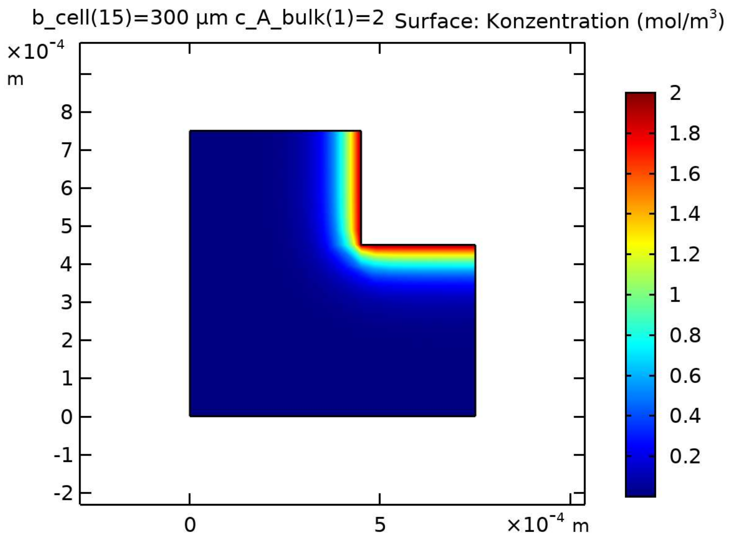

Surface: Concentration of substrate (mol/m³)

5.2.2 average concentration

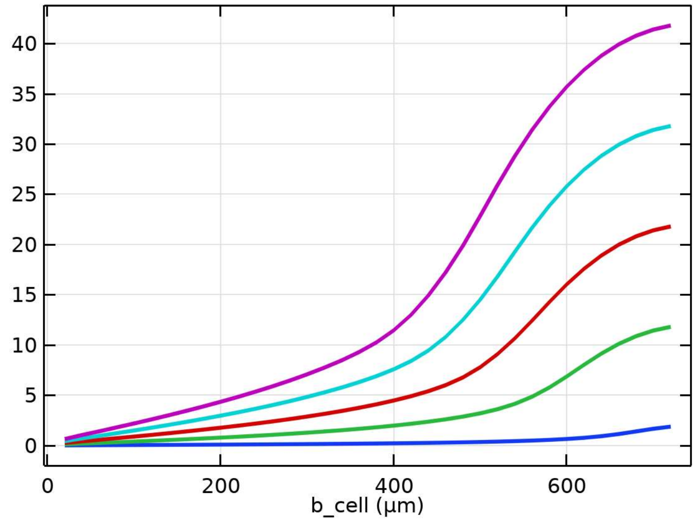

5.2.3 productivity per unit cell 1st order

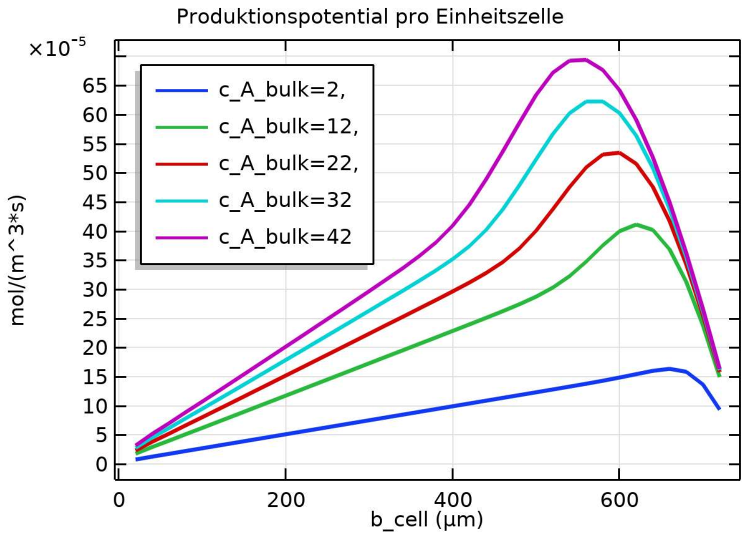

5.2.4 productivity per unit cell MM

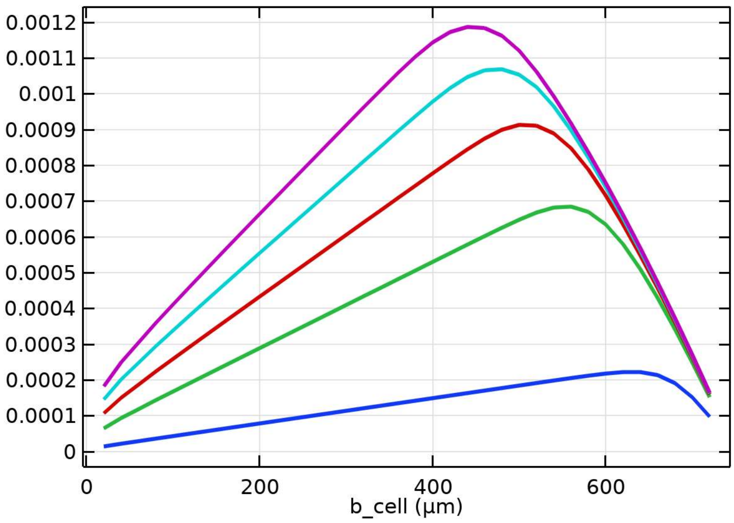

Supplement: Supplementary file 2 [file Data_Sheet_2.PDF]
