# Supplementary material for: Simulative Minimization of Mass Transfer Limitations Within Hydrogel-Based 3D-Printed Enzyme Carriers
Source: Front Bioeng Biotechnol. 2020 Apr 28;8:365. doi: 10.3389/fbioe.2020.00365 (PMC7198751; doi:10.3389/fbioe.2020.00365)

1 COMSOL MULTIPHYSICS®

Simulation report Reactor

Report date

2 Global Definitions

GLOBAL SETTINGS

Name	Reactor.mph
Version	COMSOL Multiphysics 5.4 (Build: 346)

USED PRODUCTS

COMSOL Multiphysics
Batteries & Fuel Cells Module
CAD Import Module

2.1 PARAMETERS

PARAMETER 1

Name	Expression	Value	Description
D_HG	3E-12 [m^2/s]	3E−12 m^2/s	eff. diffusivity
Km	1.4 [mmol/L]	1.4 mol/m^3	Km-value Michaelis-Menten
vmax	0.13*25 [mmol/(L*min)]	0.054167 $mol/(m^3 \cdot s)$	max. reaction rate
V_dot	0.25*3 [ml/h]	2.0833E−10 m^3/s	volumetric flow
A_Water	0.975^2 [mm^2]	9.5062E−7 m^2	cross section inlet
u_Water	V_dot/A_Water	2.1915E−4 m/s	flow velocity within the channel
c_A_bulk	2.213 [mmol/l]	2.213 mol/m^3	inlet concentration
HG_Length	10.1[mm]	0.0101 m	Length of hydrogel within reactor chamber
L_Chamber	15.8 [mm]	0.0158 m	reactor length
L_Inlet	0.5*(L_Chamber - HG_Length)	0.00285 m	length of the inlet region

3 Hydrogel reactor

3.1 GEOMETRY

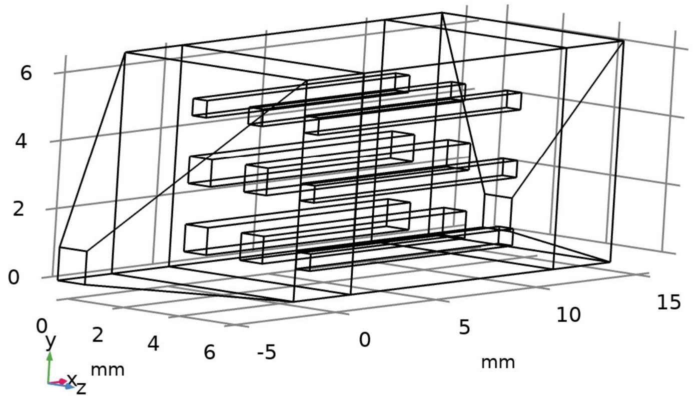

Geometry

UNITS

Length unit	mm
Angular unit	deg

3.2 TRANSPORT OF DILUTED SPECIES IN POROUS MEDIA

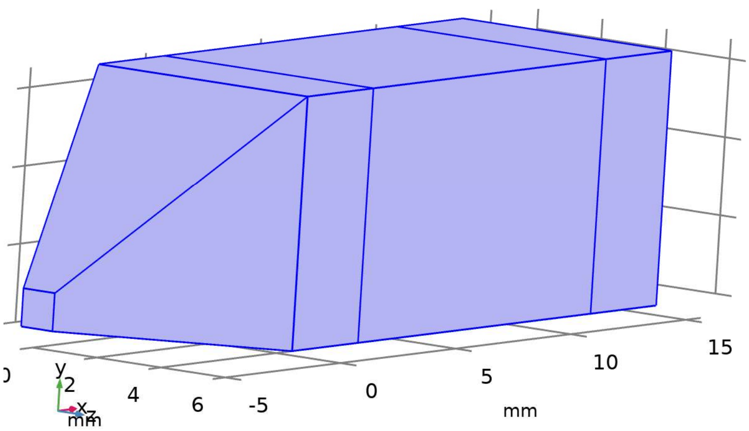

Transport of Diluted Species in Porous Media

EQUATIONS

$$\nabla \cdot \mathbf{J}_i + \mathbf{u} \cdot \nabla c_i = R_i + S_i$$
$$\mathbf{J}_i = -D_{e,i} \nabla c_i$$
$$\theta = \epsilon_p$$

FEATURES

Hydrogel Transport Properties
No Flux
Initial Values
Transport Properties Water
Symmetry
Inflow
Outflow
Reactions

3.2.1 Hydrogel Transport Properties

EQUATIONS

$$\nabla \cdot \mathbf{J}_i + \mathbf{u} \cdot \nabla c_i = R_i + S_i$$
$$\mathbf{J}_i = -D_{e,i} \nabla c_i$$
$$\theta = \epsilon_p$$

3.2.2 No Flux

EQUATIONS

$$-\mathbf{n} \cdot \mathbf{J}_i = 0$$

3.2.3 Transport Properties Water

EQUATIONS

$$\nabla \cdot \mathbf{J}_i + \mathbf{u} \cdot \nabla c_i = R_i$$
$$\mathbf{J}_i = -D_i \nabla c_i$$

3.2.4 Symmetry

EQUATIONS

$$-\mathbf{n} \cdot \mathbf{J}_i = 0$$

3.2.5 Inflow

EQUATIONS

$$c_i = c_{0,j}$$

3.2.6 Outflow

EQUATIONS

$$\mathbf{n} \cdot D_i \nabla c_i = 0$$

3.2.7 Reactions

EQUATIONS

$$\nabla \cdot \mathbf{J}_i + \mathbf{u} \cdot \nabla c_i = R_i + S_i$$

3.3 BRINKMAN EQUATIONS

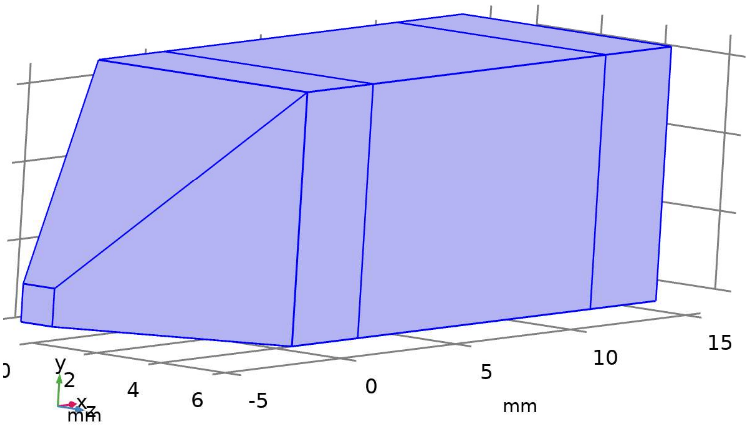

Brinkman Equations

EQUATIONS

$$\frac{1}{\epsilon_p}\rho(\mathbf{u} \cdot \nabla)\mathbf{u}\frac{1}{\epsilon_p} = \nabla \cdot [-p\mathbf{I} + \mathbf{K}] - \left(\mu\kappa^{-1} + \beta_{\mathrm{F}}|\mathbf{u}| + \frac{Q_{\mathrm{m}}}{\epsilon_p^2}\right)\mathbf{u} + \mathbf{F}$$

$$\rho\nabla \cdot (\mathbf{u}) = Q_{\mathrm{m}}$$

$$\mathbf{K} = \mu\frac{1}{\epsilon_p}\left(\nabla\mathbf{u} + (\nabla\mathbf{u})^{\mathrm{T}}\right) - \frac{2}{3}\mu\frac{1}{\epsilon_p}(\nabla \cdot \mathbf{u})\mathbf{I}$$

FEATURES

Matrix Properties
Initial Values
Wall boundary conditions
Fluid Properties
Inlet
Outlet

Symmetry

3.3.1 Matrix Properties

EQUATIONS

$$\frac{1}{\epsilon_p}\rho(\mathbf{u}\cdot\nabla)\mathbf{u}\frac{1}{\epsilon_p}=\nabla\cdot[-p\mathbf{I}+\mathbf{K}]-\left(\mu\kappa^{-1}+\beta_{\mathrm{F}}|\mathbf{u}|+\frac{Q_{\mathrm{m}}}{\epsilon_p^2}\right)\mathbf{u}+\mathbf{F}$$

$$\rho\nabla\cdot(\mathbf{u})=Q_{\mathrm{m}}$$

$$\mathbf{K}=\mu\frac{1}{\epsilon_p}\left(\nabla\mathbf{u}+(\nabla\mathbf{u})^{\mathrm{T}}\right)-\frac{2}{3}\mu\frac{1}{\epsilon_p}(\nabla\cdot\mathbf{u})\mathbf{I}$$

3.3.2 Wall boundary conditions

EQUATIONS

$$\mathbf{u}=\mathbf{0}$$

3.3.3 Fluid Properties

EQUATIONS

$$\rho(\mathbf{u}\cdot\nabla)\mathbf{u}=\nabla\cdot[-p\mathbf{I}+\mathbf{K}]+\mathbf{F}$$

$$\rho\nabla\cdot(\mathbf{u})=0$$

$$\mathbf{K}=\mu\left(\nabla\mathbf{u}+(\nabla\mathbf{u})^{\mathrm{T}}\right)$$

3.3.4 Inlet

EQUATIONS

$$\mathbf{u}=-U_0\mathbf{n}$$

3.3.5 Outlet

EQUATIONS

$$[-p\mathbf{I}+\mathbf{K}]\mathbf{n}=-\hat{p}_0\mathbf{n}$$

$$\hat{p}_0\leq p_0,$$

3.3.6 Symmetry

EQUATIONS

$$\mathbf{u}\cdot\mathbf{n}=0$$

$$\mathbf{K}_{\mathbf{n}}-(\mathbf{K}_{\mathbf{n}}\cdot\mathbf{n})\mathbf{n}=\mathbf{0},\quad \mathbf{K}_{\mathbf{n}}=\mathbf{K}\mathbf{n}$$

3.4 MESH

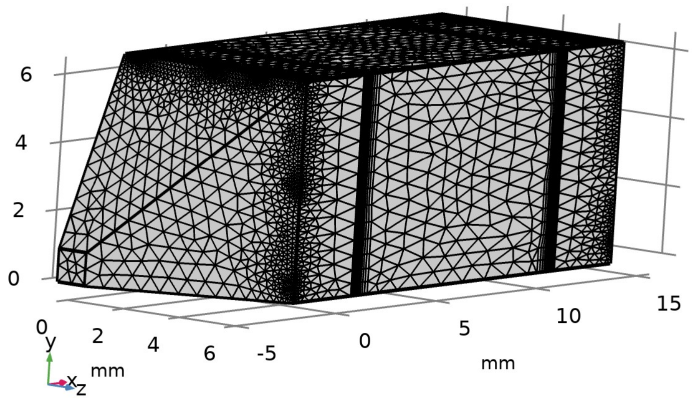

Mesh

4 hydrodynamic flow pattern

COMPUTATION INFORMATION

Computation time	8 min 33 s
CPU	Intel64 Family 6 Model 142 Stepping 9, 4 cores
Operating system	Windows 10

4.1 HYDRODYNAMIC FLOW PATTERN

STUDY SETTINGS

Description	Value
Include geometric nonlinearity	Off

MESH SELECTION

Geometry	Mesh
mesh1	mesh1

PHYSICS AND VARIABLES SELECTION

Physics interface	Discretization
Brinkman Equations (br)	physics

MESH SELECTION

Geometry	Mesh
Geometry (geom1)	mesh1

5 Enzymatic reaction stationary

COMPUTATION INFORMATION

Computation time	40 s
CPU	Intel64 Family 6 Model 142 Stepping 9, 4 cores
Operating system	Windows 10

5.1 ENZYMATIC REACTION STATIONARY

STUDY SETTINGS

Description	Value
Include geometric nonlinearity	Off

VALUES OF DEPENDENT VARIABLES

Description	Value
Settings	User controlled
Method	Solution
Study	hydrodynamic flow pattern

MESH SELECTION

Geometry	Mesh
mesh1	mesh1

PHYSICS AND VARIABLES SELECTION

Physics interface	Discretization
Transport of Diluted Species in Porous Media (tds)	physics

MESH SELECTION

Geometry	Mesh
Geometry (geom1)	mesh1

6 Enzymatic reaction dynamic

COMPUTATION INFORMATION

Computation time	17 min 30 s
CPU	Intel64 Family 6 Model 142 Stepping 9, 4 cores
Operating system	Windows 10

6.1 ENZYMATIC REACTION DYNAMIC

Times	Unit
range(0,0.25,48)	h

STUDY SETTINGS

Description	Value
Include geometric nonlinearity	Off

VALUES OF DEPENDENT VARIABLES

Description	Value
Settings	User controlled
Method	Solution
Study	hydrodynamic flow pattern

MESH SELECTION

Geometry	Mesh
mesh1	mesh1

PHYSICS AND VARIABLES SELECTION

Physics interface	Discretization
Transport of Diluted Species in Porous Media (tds)	physics

MESH SELECTION

Geometry	Mesh
Geometry (geom1)	mesh1

7 Results

7.1 DERIVED PARAMETERS

7.1.1 Product concentration effluent stationary

OUTPUT

Evaluated in	Table 5

DATA

Description	Value
Data set	Enzymatic reaction stationary/Lösung 3

EXPRESSIONS

Expression	Unit	Description
c_B	mol/m^3	Concentration

INTEGRATION SETTINGS

Description	Value
Integration order	4

7.1.2 effluent concentrations dynamic

OUTPUT

Evaluated in	Table 6

DATA

Description	Value
Data set	Enzymatic reaction dynamic/Lösung 2

EXPRESSIONS

Expression	Unit	Description
c_B	mol/m^3	Concentration of product
c_A	mol/m^3	Concentration of substrate

INTEGRATION SETTINGS

Description	Value
Integration order	4

7.1.3 Accumulation within the hydrogel

OUTPUT

Evaluated in	Table 7

DATA

Description	Value
Data set	hydrodynamic flow pattern/Lösung 1

EXPRESSIONS

Expression	Unit	Description
4	m^3	Volume of the hydrogel

INTEGRATION SETTINGS

Description	Value
Integration order	4

7.2 PLOT GROUPS

7.2.1 flow velocity pattern

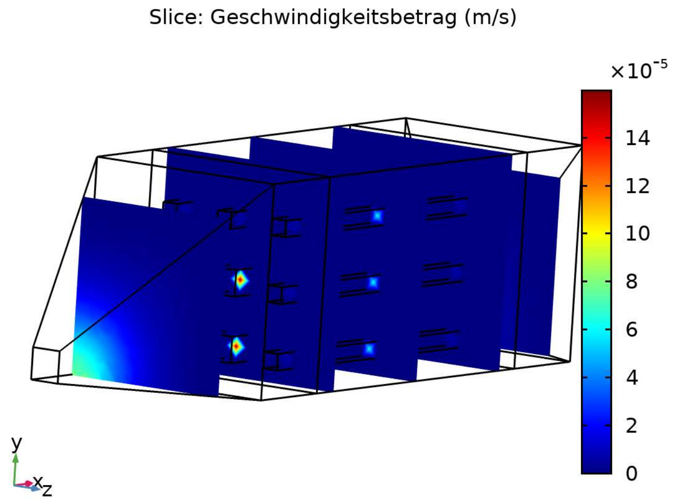

Slice: flow velocity pattern (m/s)

7.2.2 product concentration stationary

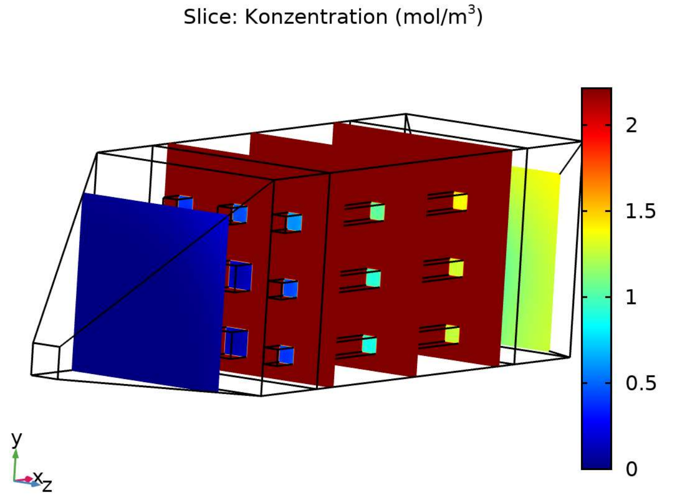

Slice: Stationary product concentration (mol/m³)

7.2.3 product concentration dynamic

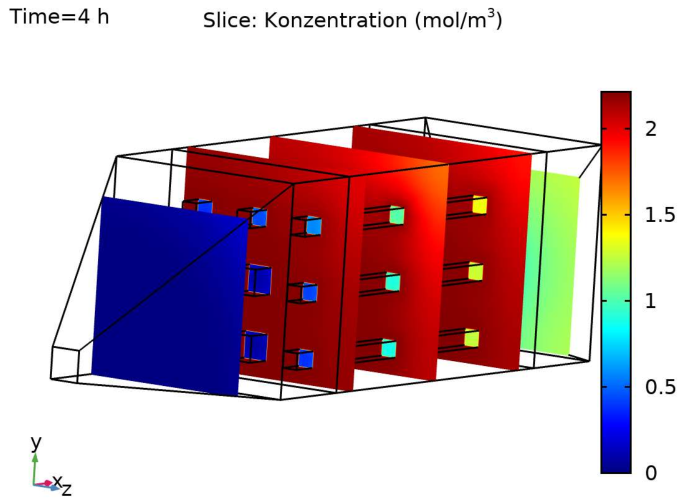

Slice: Dynamic product concentration (mol/m³)

7.2.4 effluent concentration dynamic

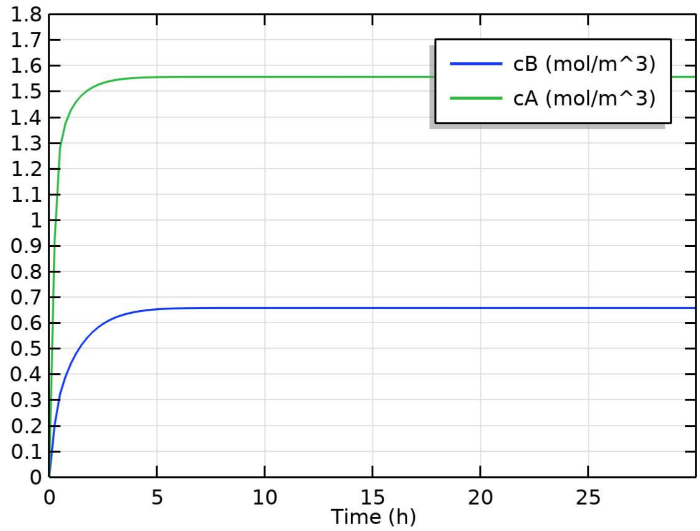

Supplement: Supplementary file 3 [file Data_Sheet_3.PDF]
