# Supplementary material for: Simulative Minimization of Mass Transfer Limitations Within Hydrogel-Based 3D-Printed Enzyme Carriers
Source: Front Bioeng Biotechnol. 2020 Apr 28;8:365. doi: 10.3389/fbioe.2020.00365 (PMC7198751; doi:10.3389/fbioe.2020.00365)

COMSOL MULTIPHYSICS®

2020 02 14 cross shape batch

1 Global Definitions

GLOBAL SETTINGS

Name	cross shape batch.mph
Version	COMSOL Multiphysics 5.4 (Build: 346)

USED PRODUCTS

COMSOL Multiphysics
Batteries & Fuel Cells Module
CAD Import Module

1.1 PARAMETERS

PARAMETERS 1

Name	Expression	Value	Description
D_HG	3E-12[m^2/s]	3E−12 m^2/s	eff. diffusivity coefficient
c_A_bulk	12[mol/m^3]	12 mol/m^3	constant boundary concentration
d_Strand	0.8E-3[m]	8E−4 m	thickness of the strand
L_Strand	3*d_Strand	0.0024 m	length of the strand
Phi	((1/2)*d_Strand)*sqrt(k_eff/D_HG)	0.92863	Thiele Modulus
V_Cross	15*d_Strand^3	7.68E−9 m^3	cross volume
vmax	0.13 [mmol/(L*min)]	0.0021667 $mol/(m^3 \cdot s)$	maximum reaction rate
Km	1.4[mmol/L]	1.4 mol/m^3	Km-value Michaelis-Menten
k_eff	vmax/(Km + c_A_bulk)	1.6169E−4 1/s	apparent reaction rate
eta	tanh(Phi)/Phi	0.78606	effectiveness factor (analytical solution)

2 Cross Shape

2.1 GEOMETRY 1

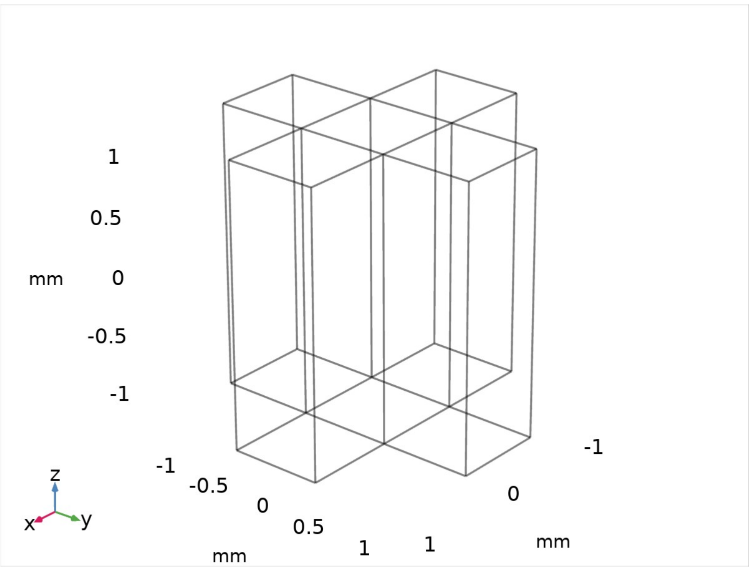

Geometry 1

UNITS

Length unit	mm
Angular unit	deg

GEOMETRY STATISTICS

**Description**	**Value**
Space dimension	3
Number of domains	1
Number of boundaries	22
Number of edges	44
Number of vertices	24

2.2 COUPLED DIFFUSION AND REACTION

USED PRODUCTS

COMSOL Multiphysics
Batteries & Fuel Cells Module

SELECTION

Geometric entity level	Domain
Selection	Domain 1

EQUATIONS

$$\mathbf{J}_i = -D_{\mathrm{e},i}\nabla c_i$$
$$\theta = \epsilon_{\mathrm{p}}$$

2.2.1 Porous Media Transport Properties

SELECTION

Geometric entity level	Domain
Selection	Domain 1

EQUATIONS

$$\mathbf{J}_i = -D_{\mathrm{e},i}\nabla c_i$$
$$\theta = \epsilon_{\mathrm{p}}$$

Matrix properties

SETTINGS

Description	Value
Porous material	Hydrogel (mat2)
Porosity	User defined
Porosity	1

Diffusion

SETTINGS

Description	Value
Fluid material	Hydrogel (mat2)
Fluid diffusion coefficient	User defined
Fluid diffusion coefficient	{{D_HG, 0, 0}, {0, D_HG, 0}, {0, 0, D_HG}}
Fluid diffusion coefficient	User defined
Fluid diffusion coefficient	{{D_HG, 0, 0}, {0, D_HG, 0}, {0, 0, D_HG}}
Effective diffusivity model	No correction

Coordinate system selection

SETTINGS

Description	Value
Coordinate system	Global coordinate system

Model input

SETTINGS

Description	Value
Temperature	User defined
Temperature	293.15[K]

2.2.2 No Flux

SELECTION

Geometric entity level	Boundary
Selection	Boundaries 1, 3–4, 7–11, 13–15, 18–19, 22

EQUATIONS

$$-\mathbf{n}\cdot(\mathbf{J}_i+\mathbf{u}c_i)=0$$

2.2.3 Initial Values

SELECTION

Geometric entity level	Domain
Selection	Domain 1

Initial values

SETTINGS

Description	Value
Concentration	{0, 0}

2.2.4 Reactions (Michaelis Menten)

SELECTION

Geometric entity level	Domain
Selection	Domain 1

EQUATIONS

$$\frac{\partial(\theta c_i)}{\partial t}+\frac{\partial(\rho c_{\mathrm{P},i})}{\partial t}+\nabla\cdot\mathbf{J}_i=\underset{\cdots}{R_i}+S_i$$

Reaction rates

SETTINGS

Description	Value
Total rate expression	User defined
Total rate expression	-((vmax*c_A)/(Km + c_A))
Total rate expression	User defined
Total rate expression	((vmax*c_A)/(Km + c_A))

Reacting volume

SETTINGS

Description	Value
Reacting volume	Total volume

2.2.5 Concentration

SELECTION

Geometric entity level	Boundary
Selection	Boundaries 2, 5–6, 12, 16–17, 20–21

EQUATIONS

$$c_i = c_{0,j}$$

Concentration

SETTINGS

Description	Value
Species c_A	On
Species c_B	On
Concentration	{c_A_bulk, 0}

Constraint settings

SETTINGS

Description	Value
Apply reaction terms on	All physics (symmetric)
Use weak constraints	Off
Constraint method	Elemental

2.3 MESH

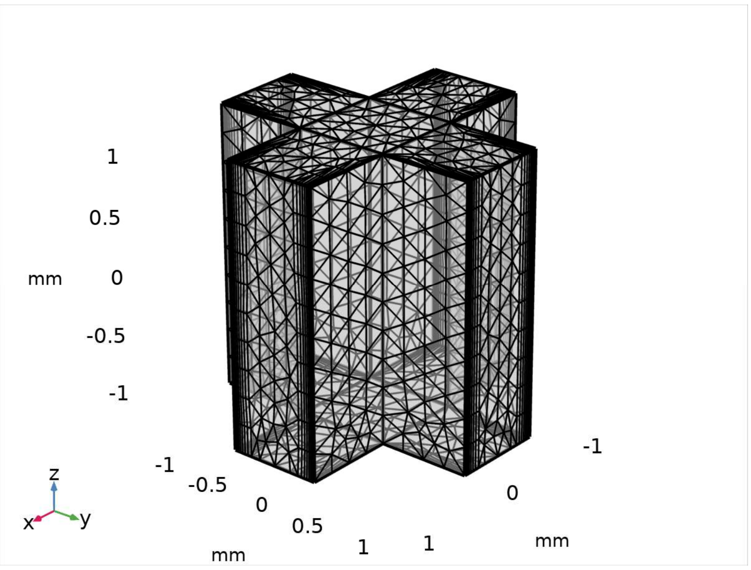

Mesh

2.3.1 Size

SETTINGS

Description	Value
Maximum element size	0.24
Minimum element size	0.0432
Curvature factor	0.6
Resolution of narrow regions	0.5
Maximum element growth rate	1.5

2.3.2 Boundary Layers 1 (bl1)

SELECTION

Geometric entity level	Domain
Selection	Geometry geom1

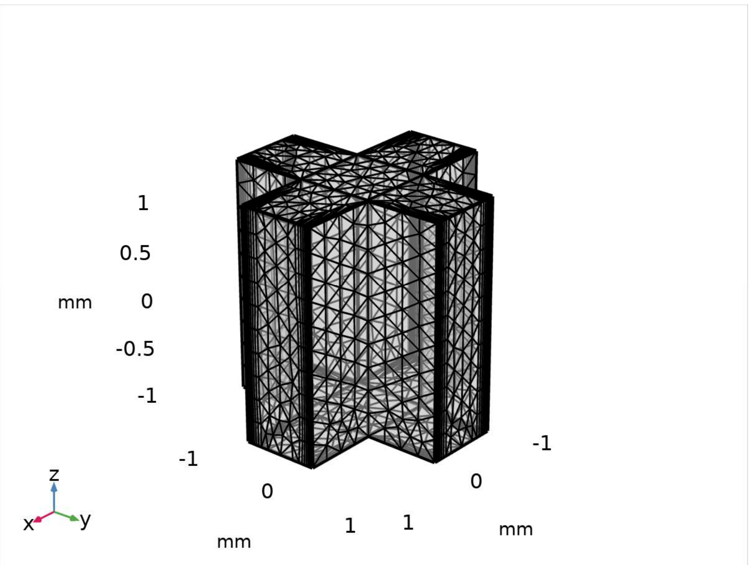

Boundary layers 1

Properties of Boundary layers (blp)

SELECTION

Geometric entity level	Boundary
Selection	Boundaries 2, 5–6, 12, 16–17, 20–21

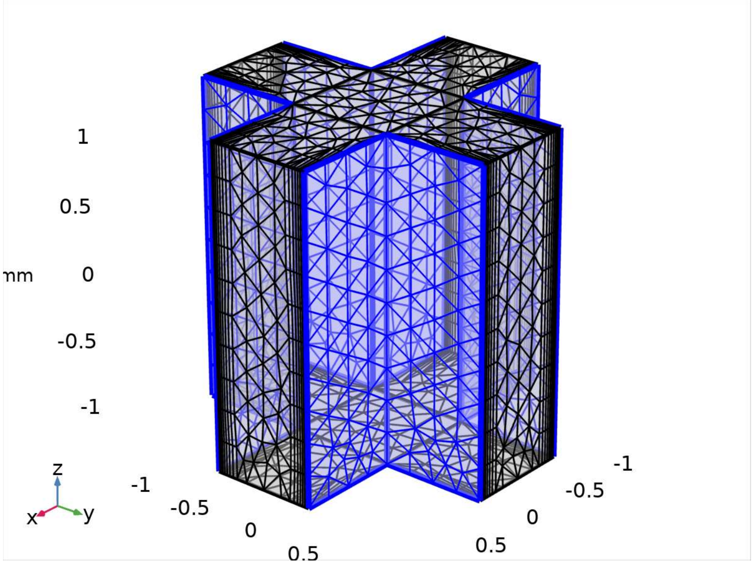

Properties of Boundary layers

SETTINGS

Description	Value
Thickness of first layer	Manual

Description	Value
Thickness	0.01

3 Study stationary

COMPUTATION INFORMATION

Computation time	3 min 16 s
CPU	Intel64 Family 6 Model 142 Stepping 9, 4 cores
Operating system	Windows 10

3.1 STATIONARY

STUDY SETTINGS

Description	Value
Include geometric nonlinearity	Off

MESH SELECTION

Geometry	Mesh
mesh1	mesh1

PHYSICS AND VARIABLES SELECTION

Physics interface	Discretization
Coupled Diffusion and Reaction (tds)	physics

MESH SELECTION

Geometry	Mesh
Geometry 1 (geom1)	mesh1

3.1.1 Study extensions

STUDY EXTENSIONS

Description	Value
Sweep type	All combinations
Run continuation for	Manual
Continuation parameter	vmax

PARAMETERS

Parameter name	Parameter value list	Parameter unit
c_A_bulk (constant boundary concentration)	0.5 2 20	mmol/L
vmax (maximum reaction rate)	10^{range(log10(0.001),1/10,log10(10))}	mmol/(L*min)

3.2 SOLVER CONFIGURATIONS

3.2.1 Solution 2

Compile Equations: Stationary (st1)

STUDY AND STEP

Description	Value
Use study	Study stationary
Use study step	Stationary

Dependent Variables 1 (v1)

GENERAL

Description	Value
Defined by study step	Stationary

INITIAL VALUE CALCULATION CONSTANTS

Constant name	Initial value source
c_A_bulk	0.5[mmol/L] 2[mmol/L] 20[mmol/L]
vmax	10^{range(log10(0.001),1/10,log10(10))}[mmol/(L*min)]

Concentration (comp1.c_A) (comp1_c_A)

GENERAL

Description	Value
Field components	comp1.c_A

Concentration (comp1.c_B) (comp1_c_B)

GENERAL

Description	Value
Field components	comp1.c_B

Stationary Solver 1 (s1)

GENERAL

Description	Value
Defined by study step	Stationary

RESULTS WHILE SOLVING

Description	Value
Probes	None

Parametric 1 (p1)

GENERAL

Description	Value
Defined by study step	Stationary
Sweep type	All combinations
Run continuation for	Manual
Continuation parameter	vmax

PARAMETERS

Parameter name	Parameter value list	Parameter unit
c_A_bulk	0.5 2 20	mmol/L
vmax	10^{range(log10(0.001),1/10,log10(10))}	mmol/(L*min)

LEAST-SQUARES DATA

Description	Value
Use least-squares parameters from file	Off

Fully Coupled 1 (fc1)

GENERAL

Description	Value
Linear solver	Iterative 1

METHOD AND TERMINATION

Description	Value
Initial damping factor	0.01
Minimum damping factor	1.0E-6
Maximum number of iterations	50

Iterative 1 (i1)

GENERAL

Description	Value
Nonlinear-based error norm	On
Maximum number of iterations	400

ERROR

Description	Value
Factor in error estimate	40

Multigrid 1 (mg1)

GENERAL

Description	Value
Use hierarchy in geometries	Geometry 1

Presmoother (pr)

SOR Line 1 (sl1)

MAIN

Description	Value
Relaxation factor	0.2

SECONDARY

Description	Value
Relaxation factor	0.4

Postsmoother (po)

SOR Line 1 (sl1)

MAIN

Description	Value
Relaxation factor	0.2

SECONDARY

Description	Value
Number of secondary iterations	2
Relaxation factor	0.4

Coarse Solver (cs)

Direct 1 (d1)

GENERAL

Description	Value
Solver	PARDISO
Pivoting perturbation	1.0E-13

4 Results

4.1 CALCULATED PARAMETERS

4.1.1 Volume integration

OUTPUT

Evaluated in	Table 1

DATA

Description	Value
Data set	Study stationary/Solution 2

EXPRESSIONS

Expression	Unit	Description
(vmax*c_A/(Km+c_A))/V_Cross	mol/(m^3*s)	production rate
c_B/V_Cross	mol/m^3	accumulated product
Phi/V_Cross	1	Thiele Modulus
(vmax*c_A/(Km+c_A))/(vmax*c_A_bulk/(Km+c_A_bulk))/V_Cross	1	effectiveness factor numerical
eta/V_Cross	1	effectiveness factor analytical

INTEGRATION SETTINGS

Description	Value
Integration order	4

4.2 PLOT GROUPS

4.2.1 effectiveness factor

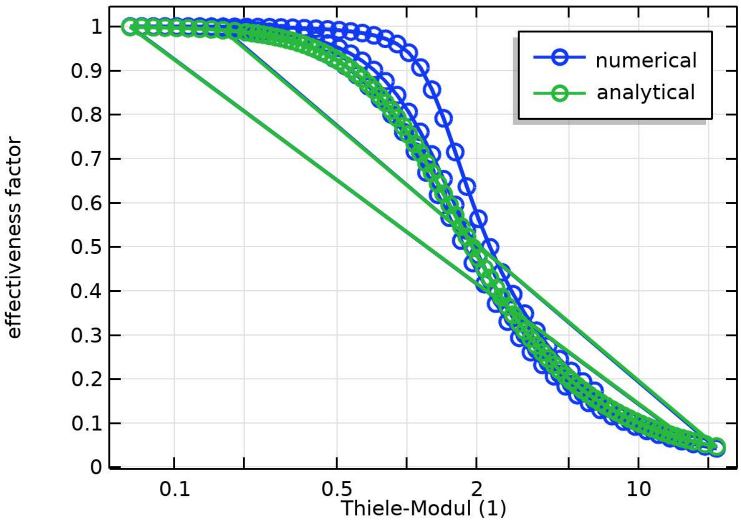

Supplement: Supplementary file 4 [file Data_Sheet_4.PDF]
